# Anthropogenic emissions shape long-term changes in PM_2.5_ concentrations and health risks in China

**DOI:** 10.1016/j.eehl.2025.100198

**Published:** 2025-11-04

**Authors:** Yiheng Wang, Guochao Chen, Yutong Yang, Zhaolei Zhang, Ruhan Zhang, Peng Wang, Hongliang Zhang

**Affiliations:** a Shanghai Key Laboratory of Atmospheric Particle Pollution and Prevention, Department of Environmental Science & Engineering, Fudan University, Shanghai 200438, China; b Department of Atmospheric and Oceanic Sciences, Fudan University, Shanghai 200438, China; c IRDR ICoE on Risk Interconnectivity and Governance on Weather/Climate Extremes Impact and Public Health, Fudan University, Shanghai 200438, China; d Institute of Eco-Chongming, Shanghai 200438, China; e Shanghai Key Laboratory of Ocean-land-atmosphere Boundary Dynamics and Climate Change, Shanghai 200438, China

**Keywords:** Fine particulate matter, Long-term, Source-specific, Pop-weighted concentration, Premature deaths

## Abstract

China has experienced an initial increase and a subsequent decrease in fine particulate matter (PM_2.5_) concentrations since the early 21st century, with substantial heterogeneity across different source contributions. This study developed a source-oriented CMAQ model to construct a source-resolved PM_2.5_ concentration database for China during 2000–2020. Subsequently, source-specific health risks and the contributions of key driving factors were systematically evaluated using the piling-up decomposition method. The results indicate that anthropogenic emissions, particularly from industrial sources, were the primary drivers of both the increase in PM_2.5_ from 2000 to 2012 (51.8%) and the subsequent decrease from 2012 to 2020 (47.6%). Currently, industrial sources remain the largest contributor to PM_2.5_ (about 32%), followed by residential (about 22%) and transportation sources (about 13%). Implementation of two-phase air pollution control measures led to a 20.4% reduction in national premature mortality attributable to PM_2.5_ from 2012 to 2020, although 10.6% of this benefit was offset by changes in population and baseline mortality rates. Throughout 2000–2020, health risks associated with anthropogenic sources consistently exceeded their proportional contribution to PM_2.5_, primarily because these emissions are concentrated in densely populated areas. These findings underscore that, in addition to implementing region-specific emission reduction policies, maintaining stringent controls on anthropogenic emissions, particularly from industrial and transportation sources, is crucial to maximizing future health benefits.

## Highlights

• Industrial activities drove the PM_2.5_ peak and subsequent decline in China.
• Industrial sources remain the largest health risk contributor currently.
• The health benefits offset by changing factors differ among various sources.
• Anthropogenic sources' health risks are disproportionate to concentrations.
• Controlling industrial and transportation emissions brings more health benefits.

## Graphical abstract

**Figure:**
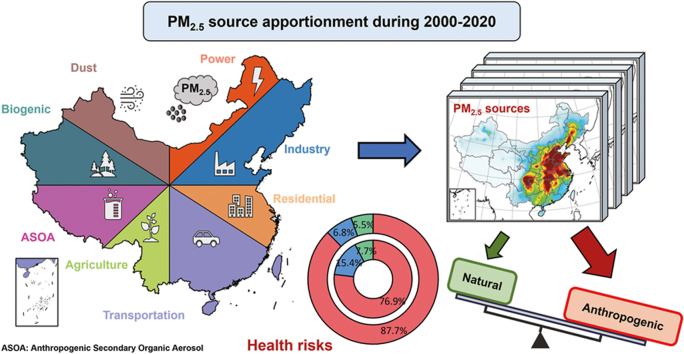

## 1 Introduction

Fine particulate matter (PM_2.5_) is one of the leading air pollutants associated with public health risks worldwide [[1], [2], [3]]. Since the early 21st century, rapid economic growth in China has increased emissions of PM_2.5_ precursors from sectors such as industry, transportation, and coal combustion, leading to widespread severe air pollution [[4], [5], [6]]. Although the implementation of stringent measures—including the *Air Pollution Prevention and Control Action Plan* (2013–2017, Phase I) and the *Three-Year Action Plan for Winning the Blue Sky Defense Battle* (2018–2020, Phase II)—has led to significant reductions in PM_2.5_ concentrations [[7], [8], [9]], reductions have been uneven across sources [10]. In addition, evolving source structures and the complexity of health risk assessment present new challenges for the next stage of air pollution control.

Accurate evaluation of policy effectiveness, quantification of population exposure and health risks, and the design of targeted mitigation strategies all require a thorough understanding of source-specific contributions to PM_2.5_ [11,12]. Considering that the ultimate goal of air pollution control is to improve public health [13], recent studies have employed chemical transport models (CTMs) and receptor-based approaches to apportion PM_2.5_-related health risks to specific emission sources [[13], [14], [15], [16], [17], [18], [19], [20], [21]]. For example, McDuffie et al. [10] and Zheng et al. [13] systematically decomposed the health risks attributable to various PM_2.5_ sources at global and national scales, respectively. Liu et al. developed a source-directional risk assessment method, revealing the critical role of coordinated controls on coal combustion, industrial, and transportation emissions in reducing PM_2.5_ health risks in Tianjin [22]. However, most existing studies are limited to relatively short time periods or specific monitoring regions and commonly rely on traditional brute-force source apportionment techniques, with insufficient consideration of dynamic factors such as meteorology, population size, and baseline mortality. Moreover, substantial differences in emission inventories, model configurations, and spatiotemporal scales across studies further constrain the comparability and generalizability of their findings. Therefore, there is a pressing need for systematic, long-term source apportionment studies that explicitly incorporate meteorology, population, and baseline mortality to robustly evaluate the health benefits of historical policies and inform more precise air pollution control strategies in the future.

In this study, we employ an improved source-oriented Weather Research and Forecasting–Community Multi-scale Air Quality (WRF–CMAQ) model to quantify the contributions of various emission sources to PM_2.5_ concentrations in China from 2000 to 2020. The integration of reactive tracer-based source apportionment overcomes the nonlinear limitations of traditional brute-force methods [23,24]. We further apply a cumulative‑decomposition approach to attribute health risks to sources and reveal heterogeneous health benefits across sources. The long-term PM_2.5_ source apportionment database established in this work provides a consistent basis for evaluating the effectiveness of historical policies and guiding future precision air pollution control strategies.

## 2 Methods

### 2.1 Model configurations

We conducted sector‑resolved source apportionment to quantify contributions of emission sectors to PM_2.5_ in each region or province. This was achieved with a modified CMAQ v5.0.2 model, which tracks sector contributions by adding reactive tracers. Compared with the brute-force method, this modification can explicitly account for nonlinear processes in atmospheric chemical reactions, thus distinguishing the final contribution of different precursor emission sources to the particulate matter concentration in a single simulation [25,26].

The WRF–CMAQ modeling domain was set up with a grid size of 197 ​× ​127 and a horizontal resolution of 36 ​km to simulate air quality in China over the 21-year period during 2000–2020 (Fig. S1, the list of cities is provided in Table S1). The final (FNL) reanalysis data from the National Centers for Environmental Prediction (NCEP), with a spatial resolution of 1.0° ​× ​1.0°, were used as meteorological inputs for WRF v4.1.2 (https://rda.ucar.edu/datasets/ds083.3/, accessed on March 2, 2023). The model was spun up during the first week of each year to minimize the impact of initial conditions. The anthropogenic emission data for China were obtained from the Multi-resolution Emission Inventory for China (MEIC v1.4) [27] (http://www.meicmodel.org/, accessed on May 15, 2024), while anthropogenic emissions for regions outside China were obtained from the Emissions Database for Global Atmospheric Research (EDGAR v5.1) (https://edgar.jrc.ec.europa.eu/, accessed on July 29, 2023) [28]. Biogenic emissions were derived from the Model of Emissions of Gases and Aerosols from Nature (MEGAN v2.1) [29]. Detailed information on the model configuration is shown in Text S1 and Tables S2 and S3.

### 2.2 Population-weighted concentration of PM_2.5_

The population-weighted ambient PM_2.5_ concentration was used to evaluate the health impact of PM_2.5_ pollution, which was calculated according to Eq. 1:

(1) $$C_{Pop}=\frac{\sum_{i}C_{i}\times Pop_{i}}{\sum_{i}Pop_{i}}$$

where, *C*_*i*_ is the simulated PM_2.5_ concentration for the cell *i*; *P*_*i*_ is the total population of the cell *i.* The gridded population data *Pop*_*i*_ were obtained from the WorldPop (https://hub.worldpop.org/project/categories?id=3, accessed on December 9, 2024).

### 2.3 Premature mortality attributable to PM_2.5_ exposure from different sources

This study estimated premature deaths attributable to PM_2.5_ exposure using the Global Exposure Mortality Model (GEMM) [30], considering mortality from both non-communicable diseases and lower respiratory infections (NCD ​+ ​LRI). For each grid cell *i*, we calculated age-specific mortality for adult populations (>25 years) in 5-year age brackets using Eqs. (2), (3).

(2) $$M_{i,t,j}=\frac{RR_{e}-1}{RR_{e}}\times Pop_{i,j,t}\times I_{j,t}$$

(3) $$RR_{e}=\exp\left\{\frac{\theta \times \ln\left[z/\left(\alpha +1\right)\right]}{1+\exp\left[-\left(z-\mu \right)/v\right]}\right\}$$

where, *RR*_*e*_ represents the relative risk (RR) of disease *e* (here referring to NCD ​+ ​LRI). *z* ​= ​max(0, *C*_*i*_ ​− ​2.4), with *θ*, *α*, *μ*, and *ν* representing dimensionless shape parameters that determine the relative risk derived from GEMM (Table S4). *C*_*i*_ represents the PM_2.5_ at grid *i*. *M*_*i,t,e*_ denotes the total number of PM_2.5_-attributable premature deaths in year *t* at grid cell *i*. Baseline mortality rates (*I*_*j,t*_) were obtained from the Global Burden of Disease Study 2021 (GBD 2021, Data Resources | GHDx, healthdata.org, accessed on September 19, 2024). Gridded population age structure data *Pop*_*i,j,t*_ were sourced from WorldPop (WorldPop:: Age and sex structures). To account for mortality estimation uncertainty, we performed 1000 Monte Carlo draws from *θ*'s normal distribution, subsequently calculating the mean mortality estimates and corresponding 95% confidence intervals.

RR provides more reliable estimates at higher PM_2.5_ mass concentrations. Due to the uncertainty in specifying the concentration ranges associated with specific sources, the health risk from various pollution sources in each grid is calculated based on the proportion of PM_2.5_ contributed by each source to the total PM_2.5_, as delineated in Eq. 4:

(4) $$M_{i,s}=M_{i}\times \left(C_{i,s}/C_{i}\right)$$

where, *M*_*i,s*_ represents the number of premature deaths associated with each source *s* at grid cell *i*. *M*_*i*_ represents the total number of premature deaths associated with PM_2.5_ exposure at grid cell *i.*

### 2.4 Isolating the factors influencing premature deaths associated with PM_2.5_ from different sources

Based on Eqs. (2), (3), premature deaths associated with different emission sources are primarily influenced by PM_2.5_ (emissions and meteorological conditions), population, and baseline mortality rate. By adopting the piling-up decomposition approach recommended by the GBD [31] and progressively adjusting various factors, the factors influencing changes in premature mortality associated with different sources between 2012 and 2020 were isolated. The specific settings for the control experiments are detailed in Table 1 and Text S2.

**Table 1:** The sensitivity experiment set up to separate the effects of population and baseline mortality rate changes on PM_2.5_-related premature deaths.

Case	Meteorology	Emissions	Population growth	Baseline mortality rate
1	2000–2020	2000–2020	2000–2020	2000–2020
2	2020	2012	2012	2012
3	2020	2020	2012	2012
4	2020	2020	2020	2012
5	2020	2020	2020	2019

### 2.5 Differences in PM_2.5_ inhalation exposure from different sources between urban and non-urban areas

The division between urban and non-urban areas in China is based on the Global Human Settlement Layer Settlement Model (GHS-SMOD) data provided by the European Commission's Global Human Settlement Layer (GHSL, https://human-settlement.emergency.copernicus.eu/download.php, accessed on December 29, 2024). This dataset classifies urban and non-urban areas at a 1 ​km resolution based on population density, proximity, and population size. The specific classification of urban and non-urban populations is provided in Text S3.

Since population exposure is directly proportional to the product of PM_2.5_ and population size, PM_2.5_ exposure is calculated as the total mass of particulate matter inhaled by the entire population over the course of a year. The PM_2.5_ exposure from different sources was then calculated using Eqs. (5), (6).

(5) $$E_{i,j,s}=C_{s}\times IR\times ET\times EF\times Pop_{i,j}$$

(6) $$E_{s}=\sum_{i}\sum_{j}E_{i,j,s}$$

where, *E*_*i,j,s*_ represents the inhalation exposure of PM_2.5_ from different sources in different age groups for each grid, *C*_*s*_ is the PM_2.5_ contributed by different sources (μg/m^3^), *IR* is the inhalation rate (m^3^/h), *ET* is the exposure time, assumed to be 24 ​h per day, and *EF* is the exposure frequency, assumed to be 365 days. The inhalation rate for each age group was compiled based on the European Chemicals Agency for individuals under 30 years old and the U.S. Environmental Protection Agency for individuals over 30 years old (Table S5). Finally, the total exposure contribution from different sources was obtained by summing the exposure across all grid points and age groups.

## 3 Results and discussion

### 3.1 Model validation

WRF meteorology and CMAQ performance were validated with data from the National Centers for Environmental Information (NCEI, https://www.ncei.noaa.gov/, accessed in March 2024) and the China National Environmental Monitoring Centre (CNEMC, http://www.cnemc.cn/, accessed on August 15, 2023). The distribution of CNEMC sites is provided in Fig. S2. Both meteorology and air‑pollution simulations agreed well with observations, with statistics within accepted ranges (Tables S6-S9).

For years before 2013, when PM_2.5_ observations were unavailable, the model results were compared with publicly available datasets [2,32,33]. Fig. S3 compares our results with three datasets for 2000–2020 and with observations for 2014–2020. Model performance is satisfactory in most regions, suggesting the general reliability of the MEIC inventory [34]. Nevertheless, the overall underestimation of annual mean concentrations may be attributed to a systematic underestimation of current emission inventories [[35], [36], [37], [38]]. In addition, the simulation results show that dust makes a significant contribution to PM_2.5_ in Northwest China (>40%, Fig. S4). Given that dust concentrations in the current model were calculated online using parameterization schemes based on land type and emission factors, considerable uncertainty remains. This may be a major reason for the underestimation of PM_2.5_ in Northwest China [[39], [40], [41]].

**Fig. 1:**
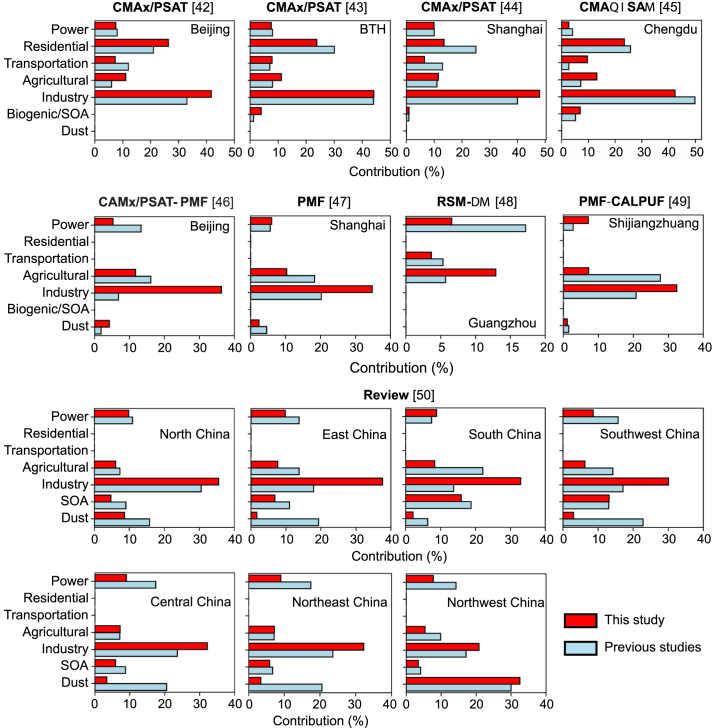
Comparison of the source apportionment of this study with the results of the previous study. The source apportionment methods used in the previous studies were labeled in each subfig, the validated regions were labeled in Fig. S1. BTH, Beijing-Tianjin-Hebei.

To verify the reliability of the source apportionment results, we extracted results for the same periods and locations and compared the source apportionment outcomes from this study with those from previous research [[42], [43], [44], [45], [46], [47], [48], [49], [50]] (Fig. 1, detailed data are provided in Table S10). Because the comparative studies are independent, differences in methods, domains and periods, meteorology, and inventories introduce variability across results, limiting the strength of this validation. Our simulated industrial contribution is slightly higher, while the contributions from transportation and dust sources are relatively lower. This is mainly due to the differences in the classifications of PM_2.5_ sources between our study and other receptor model-based studies. For example, our transportation sector includes only on‑road exhaust in MEIC, excluding aviation, shipping, and other transport‑related emissions. This is a primary reason for the underestimation of transportation contribution. In addition, dust sources in CMAQ simulations are determined based on land cover type, emission factors, and meteorological parameters, whereas dust results from receptor model-based studies may include contributions from road dust and industrial dust, potentially leading to underestimation in our results. Overall, the source apportionment results based on the MEIC inventory are reliable and show high consistency with previous CTM-based source apportionment (first row of Fig. 1). These results can support assessment of long‑term trends in PM_2.5_ source contributions in China.

### 3.2 Spatio-temporal variability in source-specific contributions

**Fig. 2:**
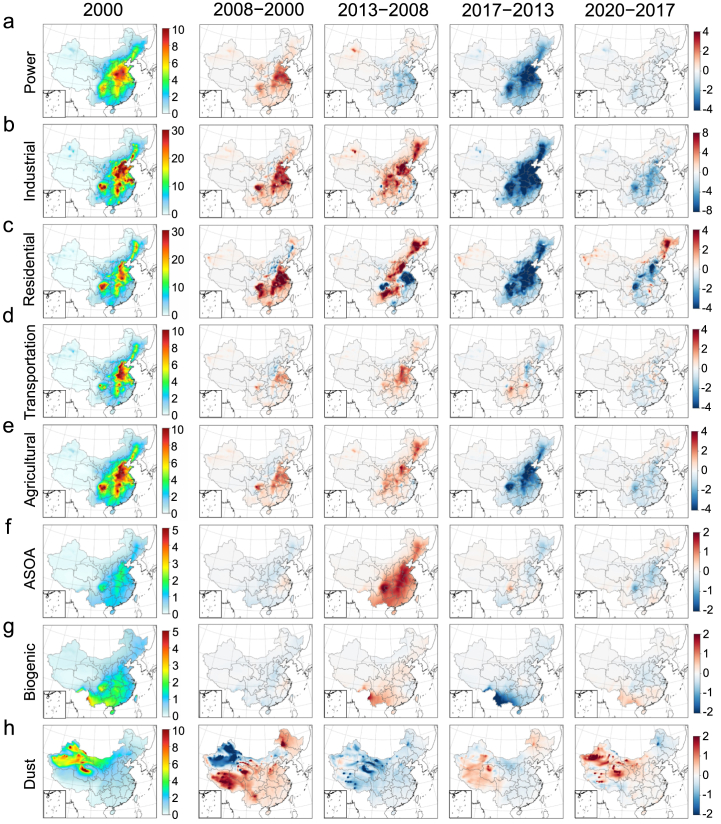
Changes in PM_2.5_ concentration contributed by (a) power, (b) industrial, (c) residential, (d) transportation, (e) agricultural, (f) Anthropogenic Secondary Organic Aerosols (ASOA), (g) biogenic, and (h) dust sources across various periods (Units: μg/m^3^). The first column represents the baseline values in 2000, and the second to fifth columns show the changes in subsequent years relative to the previous year. Standard map production based on GS (2022) 4314 with no modifications to the base map.

The contributions of different sources to PM_2.5_ display distinct spatial patterns and temporal trends (Fig. 2 and Fig. S5). Trends were determined by calculating differences in annual mean PM_2.5_ between years. Anthropogenic sources contribute significantly to PM_2.5_ in densely populated areas, largely driven by intensive human activities. Biogenic sources contribute more in South and Southwest China, while dust dominate in Northwest China. Compared to 2000, PM_2.5_ concentrations increased across most regions by 2008. This overall increase was primarily driven by the growing anthropogenic sources. Between 2008 and 2013, continued growth in anthropogenic contributions led to a further rise in PM_2.5_ levels. Following the initiation of Phase I in 2013, there was a significant decline in PM_2.5_ concentrations in China, with reductions exceeding 20 ​μg/m^3^ in East China by 2017. This decline was largely driven by substantial reductions from anthropogenic sources (Table S11 and Fig. S6). After the completion of Phase Ⅱ in 2020, PM_2.5_ concentrations further declined, though at a slower overall rate compared to the Phase Ⅰ period. Notably, residential source contributions in Northeast China even showed a slight rebound (Tables S7-S10).

Influenced by seasonal variations in human activities, meteorological conditions, and emission changes, PM_2.5_ concentrations typically peak in autumn and winter and are lower in spring and summer [51]. Source contributions also exhibit notable seasonal differences (Figs. S11-S13). Anthropogenic sources contribute less to PM_2.5_ during spring and summer, but their contributions are greater in autumn and winter, with power and residential sources exhibiting the most significant seasonal variability. Seasonal variations in natural sources also differ. During spring and autumn, dust contributions from Northwest China are particularly pronounced. In summer, higher temperatures enhance biogenic volatile organic compound (BVOC) emissions, leading to increased secondary organic aerosol (SOA) formation [52,53], particularly in South and Southwest China.

### 3.3 Source-specific contributions to population-weighted concentrations

**Fig. 3:**
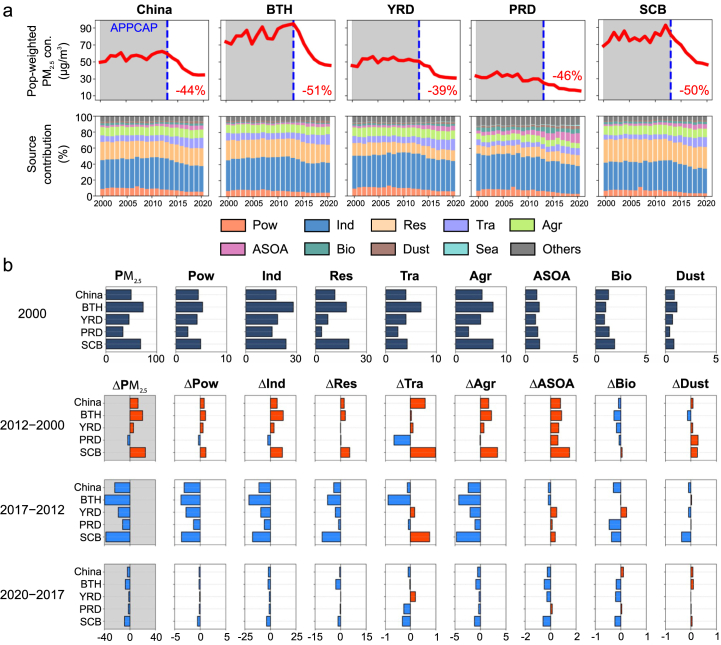
(a) Changes in population-weighted PM_2.5_ concentrations and the proportions of contributions from different sources in China and major urban agglomerations from 2000 to 2020; (b) Variations in contributions from different sources across various time periods (units: μg/m^3^). The first row represents the baseline values in 2000, and the second to fourth rows show the changes in subsequent years relative to the previous year. YRD, Yangtze River Delta; PRD, Pearl River Delta; SCB, Sichuan Basin. Pow, power generation; Ind, industry; Res, residential; Tra, transportation; Agr, agriculture; ASOA, anthropogenic secondary organic aerosol; Bio, biogenic; Dust, dust; Sea, sea salt aerosol.

Fig. 3a shows population-weighted PM_2.5_ from different sources across urban agglomerations. Industrial and residential sources were the top contributors to PM_2.5_. In the Beijing-Tianjin-Hebei (BTH) and Sichuan Basin (SCB), residential sources accounted for a higher proportion (23%–31%), primarily due to winter heating and other residential activities. In contrast, industrial sources dominated in economically developed regions such as the Yangtze River Delta (YRD) and Pearl River Delta (PRD), contributing over 40%.

Fig. 3b illustrates variations in population-weighted concentrations driven by different sources. From 2000 to 2012, the population‑weighted PM_2.5_ from anthropogenic sources, such as the power sector, industrial sources, and agricultural sources, increased nationwide. China's population-weighted average PM_2.5_ peaked in 2012, with industrial sources as the primary contributors, accounting for 51.8%. Population-weighted PM_2.5_ in the YRD and the PRD remained stable between 2000 and 2012. Source apportionment results indicate that the growth rate of anthropogenic sources in the YRD during this period was only 40% of the national average, while in the PRD, contributions from transportation, industrial, and power sources decreased by 27%, 16%, and 14%, respectively. This suggests that precursor emission controls in cities in South China were already effective before the initiation of Phase I policies.

Following the implementation of Phase Ⅰ in 2013, China's population-weighted average PM_2.5_ decreased from 62.4 ​μg/m^3^ to 38.6 ​μg/m^3^ in 2017, and to 34.6 ​μg/m^3^ by 2020. PM_2.5_ in urban agglomerations decreased by 39%–51%. During the Phase Ⅰ period, industrial sources were the primary contributors to the PM_2.5_ reductions, accounting for 46%–53% of the decline across the five urban agglomerations. However, contributions from transportation sources slightly increased in the YRD and SCB. This rise may be attributed to the growing vehicle ownership and the delayed implementation of stricter fuel standards (China V) until 2016 [54,55].

After the initiation of Phase II in 2018, population-weighted PM_2.5_ continued to decline across regions, but the rate of decrease significantly diminished, primarily due to reduced contributions from industrial sources. In addition to this, rising transportation sources in the YRD region and increases in ASOA and biogenic sources in the PRD region have partially offset the reductions in PM_2.5_ from other sources. Although PM_2.5_ in the PRD region have reached relatively low levels, the proportion of PM_2.5_ attributable to ASOA and biogenic sources has continuously risen from 7.6% in 2000 to 17.8% in 2020 (Fig. 3a). This suggests that with ongoing climate warming, the PRD region should pay closer attention to SOA contributions.

### 3.4 Premature mortality associated with different sources

**Fig. 4:**
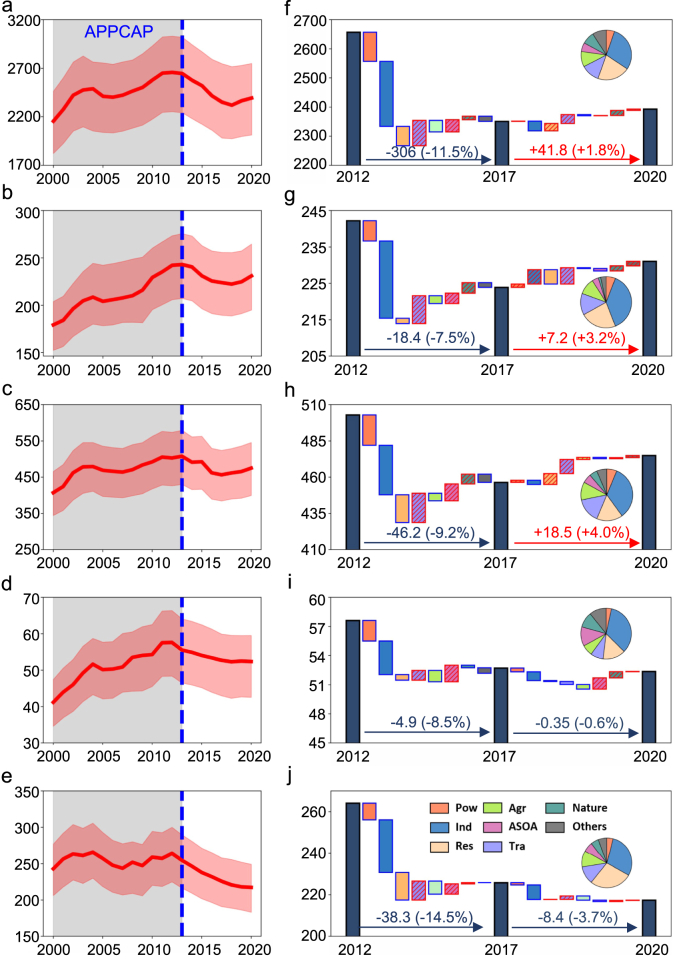
Changes in premature mortality (units: thousand) from 2000 to 2020 in (a) nationwide; (b) BTH; (c) YRD; (d) PRD; and (e) SCB. Variation in premature mortality associated with different sources between periods in (f) nationwide; (g) BTH; (h) YRD; (i) PRD, and (j) SCB. In panels (f)−(j), red borders and shading represent an increase in premature deaths compared to the previous year, whereas blue borders indicate a decrease. The pie charts show the distribution of premature deaths by different sources in 2020 as a percentage of the total premature deaths.

Fig. 4a–e illustrates trends in premature mortality related to PM_2.5_ across the nation and various urban agglomerations. In 2000, premature deaths in China due to PM_2.5_ were approximately 2155 (95% CI: 1822−2464) thousand, increasing by 23.3% to a peak of 2657 (1735−2335) thousand in 2012, before declining to 2351 (1981−2747) thousand in 2017, with a slight rise to 2393 (2013−2750) thousand from 2017 to 2020. In the BTH and YRD regions, premature mortality initially decreased during the first phase and then slightly increased during the second phase, while in the PRD and SCB regions, it continuously decreased during both phases.

The health risk changes associated with different PM_2.5_ sources during the implementation of the two-phase plan were decomposed by emission sources (Fig. 4f–j). This study grouped biogenic sources, dust sources, and sea salt aerosols as natural sources. There has been a notable decline in premature deaths attributable to industrial sources since 2012, with a 22.3% reduction nationwide from 2012 to 2017, followed by a further 4.3% reduction from 2017 to 2020. The contribution of industrial sources to premature deaths associated with PM_2.5_ dropped from 37.7% in 2012 to 31.2% in 2020 nationwide. Despite this significant decline, industrial sources remained the largest contributor to health risks in urban agglomerations by 2020, accounting for 29.4%–36.8% of premature deaths in four agglomerations. Residential sources were the second-largest contributor accounting for 22.5% of nationwide premature deaths. This was followed by transportation, agricultural, and natural sources, which contributed 12.8%, 10.5%, and 5.8% of premature deaths nationwide, respectively. Due to stringent coal-fired power plant emission policies, premature deaths from the power sector have declined annually since 2007, accounting for only 5.5% of the total in 2020.

Overall, the two-phase air pollution control plan prevented approximately 207,200 premature deaths nationwide, with about 306,000 avoided during the first phase (−11.5%) and an increase of 41,800 cases (+1.8%) during the second phase. The number of premature deaths avoided in the first phase exceeded those in the second across both the nation and various urban agglomerations. Premature deaths increased by 3.2% and 4.0% in the BTH and the YRD between 2017 and 2020, indicating that the health benefits from current emission reductions are gradually being offset by other factors. As PM_2.5_ concentrations continue to decrease, the challenges of deeper emission reductions become increasingly significant [21]. From a source perspective, except for the PRD, the rise in transportation sources was the main driver of increased premature deaths in other urban agglomerations, with a national increase of approximately 11,700 deaths associated with transportation sources since 2012. In the PRD, premature deaths attributed to ASOA and natural sources increased by 22.8% and 23.9% during this period. This highlights the need for region-specific policies that target the emissions of precursor substances from various sources.

### 3.5 Drivers of source-specific PM_2.5_ mortality

**Fig. 5:**
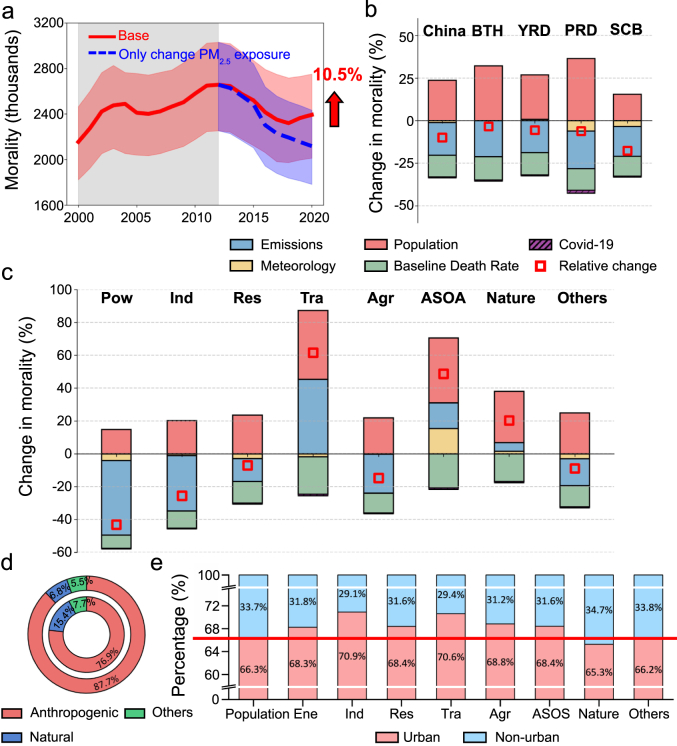
(a) Changes in premature mortality under the baseline scenario and conditions with only PM_2.5_ concentration altered. (b) Isolating the factors affecting premature mortality nationwide and in different urban agglomerations from 2012 to 2020. (c) Isolating the factors influencing premature deaths associated with different sources from 2012 to 2020. (d) The contributions of anthropogenic sources, natural sources, and other sources to PM_2.5_ concentrations (inner ring) and related health risks (outer ring) in China in 2020. (e) Differences in inhalation exposure by various sources in urban and non-urban areas in 2020. The red line indicates the baseline difference in population distribution between urban and non-urban areas; the offset of inhalation exposure from each source relative to the red line reflects the extent to which that source contributes to differences in health risk between urban and non-urban areas.

To evaluate the health benefits of actual emission reduction measures, we held population and baseline mortality rates constant at 2012 levels, while allowing annual variations in PM_2.5_. Our results show that the number of PM_2.5_-attributable premature deaths has continuously declined since 2012. By 2020, compared to the baseline scenario, premature deaths decreased by 275,000 (231,000–317,000). Overall, from 2012 to 2020, stringent emission controls led to a 20.4% decrease in health risks associated with PM_2.5_ exposure. However, changes in other factors, including population growth, aging, and declining baseline mortality rates, led to a 10.5% increase that partially offset the health benefits (Fig. 5a).

We further applied a cumulative decomposition approach to disentangle the contributions of different factors from regional and source-specific perspectives (Fig. 5b and c). Meteorological changes contributed marginally to the decrease in PM_2.5_ (1.2% nationwide). In contrast, population growth and aging increased premature deaths by 23.7%. The decline in baseline mortality rates contributed to a 12.7% reduction in premature deaths. Furthermore, the potential influence of COVID-19 in 2020 on baseline mortality rates was isolated, indicating a minimal effect on PM_2.5_-related health risks (0.54% nationwide). During this period, except in SCB, the population became more concentrated in urban agglomerations (Fig. S14), leading to a more pronounced increase in premature deaths attributable to population changes in urban areas. In the BTH, YRD, and PRD, population growth and aging led to increases in premature deaths ranging from 26.1% to 36.4%.

After 2012, health risks associated with power, industrial, residential, and agricultural sources declined, primarily due to effective emission controls. In contrast, health risks attributable to transportation sources, ASOA, and natural sources increased. Notably, transportation-related health risks increased the most, rising by 61.5% from 2012 to 2020, followed by ASOA-related risks, which rose by 48.7%. These trends largely reflect the limited effectiveness of emission controls targeting these sources, underscoring the urgent need for more stringent precursor emission controls for transportation and ASOA sources in the future. In 2020, anthropogenic, natural, and other sources contributed 76.9%, 15.4%, and 7.7% to total PM_2.5_, but 87.7%, 6.8%, and 5.5% to associated health risks, respectively (Fig. 5d). Similar results were observed each year from 2000 to 2020 (Fig. S15), highlighting that anthropogenic PM_2.5_ remains the dominant contributor to health risks, both historically and currently. This likely reflects the higher density of anthropogenic emissions in populous urban regions, resulting in greater exposure among urban populations [56,57]. To further elucidate this phenomenon, we classified urban and non-urban areas in China based on the GHS-SMOD model and calculated source-specific inhalation exposures in these regions (Fig. 5e). In 2020, urban and non-urban populations accounted for 66.3% and 33.7% of the national population, respectively. The relative difference in inhalation exposure to anthropogenic PM_2.5_ between urban and non-urban areas exceeded the population ratio, indicating that urban populations bear a disproportionately higher burden of anthropogenic PM_2.5_ exposure [58]. Notably, the shares of industrial and transportation-related inhalation exposures in urban areas exceeded 70%, suggesting that prioritizing emission controls on these sources would yield the greatest health benefits. In contrast, natural sources contributed more to exposure in non-urban areas (34.7%). As urbanization progresses, more people will migrate to high-exposure urban areas, emphasizing the urgent need for stricter emission reduction strategies for all anthropogenic sources, especially industrial and transportation sources.

### 3.6 Uncertainty analysis

This study has some uncertainties and limitations. First, the uncertainty in the simulated PM_2.5_ in this study mainly arises from the modeling errors of nitrate and SOA. Previous studies have shown that the inadequate representation of heterogeneous reactions of N_2_O_5_ and NO_2_ are the primary reason for the underestimation of particulate nitrate, leading to an underprediction of nighttime NO_3_ formation [59]. In addition, the underestimation of NH_3_ emissions in the MEIC inventory (about 27% nationwide) and the underestimation of sulfate further suppresses nitrate formation [[60], [61], [62]]. Uncertainty in SOA primarily stems from incomplete precursor emission inventories, errors in the MEGAN model, and incomplete SOA formation mechanisms [63]. Furthermore, inaccuracies in vegetation type or distribution and insufficient consideration of various anthropogenic VOCs with different volatilities can also lead to SOA underestimation [[63], [64], [65]]. Although the CMAQ model mechanism employed in this study incorporates improved multiphase formation pathways, effectively narrowing the gap between simulated and observed SOA [66], we recommend that future work further improve the parameterization of these key processes and strengthen constraints from local observations and emission inventories to enhance simulation accuracy.

Second, the source apportionment method in this study relies on the MEIC emission inventory. Although model validation results fall within acceptable ranges, uncertainties in precursor emissions in the inventory inevitably affect the performance of source apportionment. Moreover, due to the lack of source-specific exposure–response relationships, we assumed equal toxicity of PM_2.5_ from different sources to ensure comparability in assessing exposure disparities. However, some studies have demonstrated that PM_2.5_ from different sources may have distinct toxicity. For example, emissions from coal-fired power plants and transportation have been shown to pose significantly higher health risks compared to other sources [20,67,68]. A cohort study in the U.S. by Henneman et al. reported that PM_2.5_ from coal-fired power plants had a relative risk 2.1 times higher than that from any other source in the country [69]. Pye et al. found that in the U.S., SOA was most strongly associated with cardiorespiratory disease mortality, with a health hazard significantly greater than that of total PM_2.5_ [70]. Unfortunately, China currently lacks longitudinal cohort studies on long-term exposure to source-specific PM_2.5_, making it difficult to quantify the relative risks associated with different sources. This gap may lead to the under- or overestimation of health risks related to specific sources.

Finally, this study distinguished between urban and rural areas using the GHS-SMOD dataset, aiming to reflect exposure differences among populations in different regions. Previous research has shown that rural-to-urban migration is often accompanied by a shift to cleaner fuels, thereby reducing emissions and yielding health benefits [71]. However, the emission estimates in this study, based on the MEIC inventory, did not account for changes in emissions following urban migration. Additionally, when calculating inhalation exposure, we resampled CMAQ, population, and GHS-SMOD urban–rural classification data onto a common grid, which limited our ability to perform higher-resolution exposure analyses. This underscores the need to develop higher-resolution models.

## 4 Conclusions

This study used a source-oriented CMAQ model to analyze PM_2.5_ source contributions and related health risks across China during 2000–2020. Our work provides essential data for long-term, large-scale assessments and offers important scientific support for more targeted air pollution control and health policy. The findings reveal that reductions in premature mortality under China's two-phase air pollution control policies varied significantly by region. As PM_2.5_ declined, the relative contributions of transportation sources in the BTH and YRD regions, and SOA in the PRD, increased. This highlights new challenges for air quality management as emission controls deepen. Using a piling-up decomposition method, we further disentangled the impacts of meteorology, emissions, population structure, and baseline mortality on source-specific PM_2.5_ health burdens, showing that demographic changes can partially offset the health benefits from emission reductions. Moreover, our findings emphasize that the health burden attributable to anthropogenic sources remains disproportionately high relative to their contribution to overall PM_2.5_, primarily because urban residents are exposed to much higher levels of anthropogenic PM_2.5_, especially from industrial and transportation sources, which account for over 70% of urban exposure. These results underscore the need for sustained, stringent, and region-specific precursor emission controls, particularly targeting industrial and transportation sources, to maximize public health benefits.

## CRediT authorship contribution statement

**Yiheng Wang:** Writing – original draft, Visualization, Software, Formal analysis, Data curation. **Guochao Chen:** Software, Formal analysis, Data curation. **Yutong Yang:** Software, Data curation. **Zhaolei Zhang:** Software, Data curation. **Ruhan Zhang:** Validation, Software, Data curation. **Peng Wang:** Methodology, Data curation, Conceptualization. **Hongliang Zhang:** Writing – review & editing, Supervision, Resources, Methodology, Investigation, Conceptualization.

## Declaration of competing interests

The authors declare that they have no known competing financial interests or personal relationships that could have appeared to influence the work reported in this paper.
