# Supplementary material for: Anthropogenic emissions shape long-term changes in PM2.5 concentrations and health risks in China
Source: Eco Environ Health. 2025 Nov 4;4(4):100198. doi: 10.1016/j.eehl.2025.100198 (PMC12664979; doi:10.1016/j.eehl.2025.100198)
Supplement: Multimedia component 1 [file mmc1.docx]

*Supplementary Material of*

**Anthropogenic emissions shape long-term changes in PM_2.5_ concentrations and health risks in China**

Yiheng Wang^a^, Guochao Chen^a^, Yutong Yang^a^, Zhaolei Zhang^a^, Ruhan Zhang^a^, Peng Wang^b,c,e^, Hongliang Zhang^a,c,d, *^

^a^ Shanghai Key Laboratory of Atmospheric Particle Pollution and Prevention, Department of Environmental Science & Engineering, Fudan University, Shanghai 200438, China

^b^ Department of Atmospheric and Oceanic Sciences, Fudan University, Shanghai 200438, China

^c^ IRDR ICoE on Risk Interconnectivity and Governance on Weather/Climate Extremes Impact and Public Health, Fudan University, Shanghai 200438, China

^d^ Institute of Eco-Chongming, Shanghai 200438, China

^e^ Shanghai Key Laboratory of Ocean-land-atmosphere Boundary Dynamics and Climate Change, Shanghai 200438, China

***Corresponding Author.**

**Email:** zhanghl@fudan.edu.cn (H. Zhang)

**Contents**

**Summary:** The supplemental material is 36 pages, including 3 Texts, 11 tables and 15 figures.

**Text：**

**Text. S1.** Classification of PM_2.5_ Sources

**Text. S2.** The detailed steps of the piling-up decomposition approach

**Text. S3.** Classification of urban and non-urban populations in China based on the Global Human Settlement Layer Settlement Mode (GHS-SMOD)

**Tables：**

**Table S1.** List of cities included in the agglomeration

**Table S2.** WRF scheme set-up

**Table S3.** WRF-CMAQ Model configurations

**Table S4.** Global Exposure Mortality Model (GEMM) parameter estimates

**Table S5.** Long-term inhalation rate used in this study based on European Chemicals Agency (ECHA) and US Environmental Protection Agency (EPA)

**Table S6.** Meteorology performance in all the months from 2000 to 2020

**Table S7.** Model performance on PM_2.5_, in January to December from 2014 to 2020

**Table S8.** Model performance on PM_2.5_ from 2014 to 2020 in different regions.

**Table S9.** Verification of CMAQ simulation results for urban and non-urban areas

**Table S10.** Comparison of results from previous source resolution studies with our simulation results

**Table S11.** Anthropogenic emission reduction measures taken by China during the two-phase air pollution prevention and control program

**Figures:**

**Fig. S1** Study domain and validation region divisions.

**Fig. S2.** Distribution of China National Environmental Monitoring Center (CNEMC) Monitoring Sites

**Fig. S3** Comparison of CMAQ simulation results with publicly available datasets

**Fig. S4.** Proportion of PM_2.5_ contributed by dust in the total concentration during 2000-2020

**Fig. S5.** Changes in the total PM_2.5_ concentrations and those contributed by sea salt and other sources across different periods

**Fig. S6. Changes in precursor emissions from different sources in China (2000–2020) based on MEICv1.4**

**Fig. S7.** Changes in anthropogenic NH_3_ emissions between different time periods

**Fig. S8.** Changes in anthropogenic NOx emissions between different time periods

**Fig. S9.** Changes in anthropogenic PM_2.5_ emissions between different time periods

**Fig. S10.** Changes in anthropogenic SO_2_ emissions between different time periods

**Fig. S11.** Seasonal variations in PM_2.5_ concentrations during 2000-2020

**Fig. S12.** Seasonal variations in PM_2.5_ concentrations contributed by anthropogenic sources during 2000**–**2020

**Fig. S13.** Seasonal variations in PM_2.5_ concentrations contributed by nature and others sources during 2000**–**2020

**Fig. S14.** Population Changes in China between 2012 and 2020

**Fig. S15.** Contribution ratios of anthropogenic sources, natural sources, and other sources to PM_2.5_ concentrations and related health risks in China from 2000 to 2020.

**Text S1.** Classification of PM_2.5_ Sources.

In this study, PM_2.5_ sources were categorized based on emission inventories and generation processes. The emission inventory provides detailed descriptions of each sector: the power generation sector (Pow) refers to emissions from coal-fired power plants, while the industrial sector (Ind) covers emissions from stationary industrial facilities and industrial processes. Residential sector (Res) emissions include fossil fuels for energy as well as biofuels for heating and cooking. The transportation sector (Tra) includes exhaust and evaporative emissions from on-road vehicles, and agricultural emissions (Agr) are primarily ammonia emissions associated with livestock and fertilizer use. Natural sources of PM_2.5_ include biogenic emissions from the Model of Emissions of Gases and Aerosols from Nature (MEGAN2.1)^[1]^, wind-blown dust, and sea salt aerosol emissions. This model relies on key input data to estimate biogenic emissions, particularly Plant Functional Type (PFT) and Leaf Area Index (LAI) data. They are derived from MCD12Q1 (interannual land cover data, 500m resolution, including PFT classification, [https://e4ftl01.cr.usgs.gov/MOLT/](https://e4ftl01.cr.usgs.gov/MOLT/)) and MOD15A2H (8-day resolution, [https://e4ftl01.cr.usgs.gov/MOTA/](https://e4ftl01.cr.usgs.gov/MOTA/)), respectively. For the Plant Functional Type (PFT) data, this study updates the dataset annually to reflect interannual changes in vegetation types. For the Leaf Area Index (LAI) data, we update the dataset every 8 days to capture the temporal variation of leaf area index within each year. Dust and sea salt emissions were generated in line during simulations. The simulated secondary organic aerosols (SOA) were further divided into anthropogenic secondary organic aerosols (ASOA) and biogenic secondary organic aerosols (BSOA).

**Text S2.** The detailed steps of the piling-up decomposition approach.

The source apportionment of mortality changes for the 2012**–**2020 was accomplished by controlling the relevant factors one by one, following the decomposition approach proposed by GBD 2015. For each specific source and urban agglomeration, we calculated the percentage contributions of four independent factors to the changes in excess mortality rates: (1) changes in ambient PM_2.5_ concentrations, (2) population changes, (3) changes in baseline mortality rates (The baseline mortality rate of 2019 was used to represent the normal variation in baseline mortality), and (4) changes in baseline mortality rates caused by the 2020 Covid-19 pandemic. The specific formula (1-9) is shown as follows:

$M_{2012}=\sum_{\text{e}} \sum_{i} y_{e}^{2012} \frac{RR_{e}^{2012met+2012emis}-1}{RR_{e}^{2012met+2012emis}}Pop_{i}^{2012}$ (1)

$M_{2020}=\sum_{\text{e}} \sum_{i} y_{e}^{2020} \frac{RR_{e}^{2020met+2020emis}-1}{RR_{e}^{2020met+2020emis}}Pop_{i}^{2020}$ (2)

$A=\sum_{\text{e}} \sum_{i} y_{e}^{2012} \frac{RR_{e}^{2020met+2012emis}-1}{RR_{e}^{2020met+2012emis}}Pop_{i}^{2012}$ (3)

$B=\sum_{\text{e}} \sum_{i} y_{e}^{2012} \frac{RR_{e}^{2020met+2020emis}-1}{RR_{e}^{2020met+2020emis}}Pop_{i}^{2012}$ (4)

$C=\sum_{\text{e}} \sum_{i} y_{e}^{2012} \frac{RR_{e}^{2020met+2020emis}-1}{RR_{e}^{2020met+2020emis}}Pop_{i}^{2020}$ (5)

$D=\sum_{\text{e}} \sum_{i} y_{e}^{2019} \frac{RR_{e}^{2020met+2020emis}-1}{RR_{e}^{2020met+2020emis}}Pop_{i}^{2020}$ (5)

$\text{Meteorology contribution (\%) = }\frac{A-M_{2012}}{M_{2012}}\times100\%$ (6)

$\text{Emission contribution (\%) = }\frac{B-A}{M_{2012}}\times100\%$ (7)

$\text{Population change (\%) = }\frac{C-B}{M_{2012}}\times100\%$ (8)

$\text{Baseline death rate change (\%) = }\frac{D-C}{M_{2012}}\times100\%$ (9)

$\text{Covid-19 change (\%) = }\frac{M_{2020}-D}{M_{2012}}\times100\%$ (10)

**Text S3.** Classification of urban and non-urban populations in China based on the Global Human Settlement Layer Settlement Mode (GHS-SMOD)

GHS-SMOD ([Global Human Settlement - Download - European Commission, copernicus.eu](https://human-settlement.emergency.copernicus.eu/download.php?ds=smod), last accessed on 29 December 2024) classifies the land surface into 1-kilometer grids using the "urbanization hierarchy" method recommended by the United Nations and the European Union. This classification is based on population density, proximity, and population size, dividing areas into 8 categories at the L2 level, primarily consisting of two major types:

Urban areas:

1. Urban Centre (UC): Population density ≥ 1500 persons/km² and total population of the agglomeration ≥ 50,000; typically, the core area of large cities.
2. Dense Urban Cluster (DUC): Population density ≥ 1500 persons/km² and agglomeration population between 5000 and 50,000; referring to small and medium-sized cities or high-density extension areas of large cities.
3. Semi-dense Urban Cluster (SDUC): Population density ≥ 900 persons/km², agglomeration population ≥ 2500, and buffered from dense urban clusters or urban centers.
4. Suburban or Peri-urban Area (SU): Belonging to urban agglomerations but not included in the above three categories, with population density ≥ 300 persons/km² and agglomeration population ≥ 5000.

Non-urban areas：

1. Rural Cluster (RC): Population density ≥ 300 persons/km² and agglomeration population between 500 and 5,000.
2. Low Density Rural Grid Cell (LDR): Population density ≥ 50 persons/km² and not classified as a Rural Cluster.
3. Very Low-Density Rural Grid Cell (VLDR): Population density < 50 persons/km².

The raster data (1 km resolution) of the above regional classifications and population data were resampled to the coordinate system and resolution of the CMAQ grid. For each CMAQ grid cell, the area proportion of each GHS-SMOD type within it was statistically calculated, and then the total urban and non-urban population in each grid was obtained through the area, as shown in Equation (9):

$Pop_{type}=f_{type}\times Pop_{total}$ (11)

Where *f_type_* is the proportion of the area of the type (urban or non-urban) in grid cell *i*. Urban population is derived from categories 1, 2, 3, and 4, while non-urban population is obtained from categories 5, 6, and 7.

**Table S1.** List of cities included in the agglomeration.

| Agglomeration | Cities |
| --- | --- |
| Beijing-Tianjin-Hebei  (BTH) | Beijing; Tianjin; Shijiazhuang; Tangshan; Qinhuangdao; Handan; Xingtai; Baoding; Zhangjiakou; Chengde; Cangzhou; Langfang; Hengshui |
| Yangtze River Delta  (YRD) | Shanghai; Nanjing; Wuxi; Xuzhou; Changzhou; Suzhou (Jiangsu); Nantong; Lianyungang; Huai’an; Yancheng; Yangzhou; Zhenjiang; Taizhou (Jiangsu); Suqian; Hangzhou; Ningbo; Wenzhou; Jiaxing; Huzhou; Shaoxing; Jinhua; Quzhou; Zhoushan; Taizhou (Zhejiang); Lishui; Hefei; Wuhu; Bengbu; Huainan; Ma'anshan; Huaibei; Tongling; Anqing; Huangshan; Chuzhou; Fuyang; Suzhou (Anhui); Chaohu; Xuancheng; Lu'an; Bozhou; Chizhou; Huaibei. |
| Peral River Delta  (PRD) | Guangzhou; Shenzhen; Zhuhai; Foshan; Dongguan; Zhongshan; Jiangmen; Huizhou; Zhaoqing. |
| Sichuan Basin  (SCB) | Chengdu; Deyang; Mianyang; Guangyuan; Suining; Neijiang; Leshan; Meishan; Zigong; Yibin; Luzhou; Ziyang; Chongqing |

**Table S2.** WRF scheme set-up

| Microphysics | Thompson |
| --- | --- |
| Longwave Radiation | RRTM |
| Shortwave Radiation | Goddard |
| Surface Layer | Monin-Obukhov |
| Land Surface | Noah |
| Cumulus Parameterization | Grell-Devenyi |
| Planetary Boundary layer | YSU |

**Table S3.** WRF-CMAQ Model configurations

| Simulation time | 2000**–**2020 |
| --- | --- |
| Chemical transport model | CMAQ v5.0.2 |
| meteorological model | WRF v4.1.2 |
| resolution | 36 km × 36 km |
| chemical mechanism | SAPRC99 |
| aerosol mechanism | AERO6 |
| Meteorological inputs | FNL |
| Anthropogenic emissions | MEIC v1.4 (China) and  Edgar v5.1 (outside China) |
| Biogenic emissions | MEGAN 2.1 |

**Table S4.** Global Exposure Mortality Model (GEMM) parameter estimates[2]

| Cause of Death | Age Range (years) | θ | standard error θ | α | μ | ν |
| --- | --- | --- | --- | --- | --- | --- |
| NCD+LRI | 25+ | 0.1430 | 0.01807 | 1.6 | 15.5 | 36.8 |
| NCD+LRI | 25−29 | 0.1585 | 0.01477 | 1.6 | 15.5 | 36.8 |
| NCD+LRI | 30−34 | 0.1577 | 0.01470 | 1.6 | 15.5 | 36.8 |
| NCD+LRI | 35−39 | 0.1570 | 0.01463 | 1.6 | 15.5 | 36.8 |
| NCD+LRI | 40−44 | 0.1558 | 0.01450 | 1.6 | 15.5 | 36.8 |
| NCD+LRI | 45−49 | 0.1532 | 0.01425 | 1.6 | 15.5 | 36.8 |
| NCD+LRI | 50−54 | 0.1499 | 0.01394 | 1.6 | 15.5 | 36.8 |
| NCD+LRI | 55−59 | 0.1462 | 0.01361 | 1.6 | 15.5 | 36.8 |
| NCD+LRI | 60−64 | 0.1421 | 0.01325 | 1.6 | 15.5 | 36.8 |
| NCD+LRI | 65−69 | 0.1374 | 0.01284 | 1.6 | 15.5 | 36.8 |
| NCD+LRI | 70−74 | 0.1319 | 0.01234 | 1.6 | 15.5 | 36.8 |
| NCD+LRI | 75−79 | 0.1253 | 0.01171 | 1.6 | 15.5 | 36.8 |
| NCD+LRI | 80+ | 0.1141 | 0.01071 | 1.6 | 15.5 | 36.8 |

**Table S5.** Long-term inhalation rate used in this study based on European Chemicals Agency (ECHA) and US Environmental Protection Agency (EPA)[3].

| Age group | Inhalation rates (m^3^/24-hour day) |
| --- | --- |
| 0 to < 5 years | 8.9 |
| 5 to < 10 years | 12.0 |
| 10 to < 15 years | 15.2 |
| 15 to < 20 years | 16.3 |
| 20 to < 30 years | 15.7 |
| 30 to < 40 years | 16.0 |
| 40 to < 50 years | 16.0 |
| 50 to < 60 years | 15.7 |
| 60 to < 65 years | 14.2 |
| ≥ 65 years | 12.9 |

**Table S6.** Meteorology performance in all the months from 2000 to 2020 (OBS is mean observation; PRE is mean prediction; MB is mean bias; GE is gross error; RMSE is root mean square error). The benchmarks are suggested by Emery, et al. [4]. The values that do not meet the criteria are denoted in the bold.

|  |  | 2000 | | 2001 | | | 2002 | | | 2003 | | | 2004 | | | 2005 | | | 2006 | | | 2007 | | | 2008 | | 2009 | | 2010 | | | Criteria |  |
| --- | --- | --- | --- | --- | --- | --- | --- | --- | --- | --- | --- | --- | --- | --- | --- | --- | --- | --- | --- | --- | --- | --- | --- | --- | --- | --- | --- | --- | --- | --- | --- | --- | --- |
| T2 (K) | OBS | 287.08 | 285.75 | | | 286.00 | | | 285.77 | | | 287.76 | | | 285.70 | | | 285.18 | | | 286.51 | | | 287.73 | | | 286.17 | | | 286.32 | |  |  |
|  | PRE | 286.25 | 284.97 | | | 285.01 | | | 285.21 | | | 287.05 | | | 284.94 | | | 284.44 | | | 285.77 | | | 287.15 | | | 285.55 | | | 285.63 | |  |  |
|  | MB | **-0.82** | | **-0.77** | | | **-0.98** | | | **-0.55** | | | **-0.69** | | | **-0.74** | | | **-0.72** | | | **-0.71** | | | **-0.57** | | **-0.59** | | **-0.66** | | | \|error\| ≤ 0.5 |  |
|  | RMSE | 3.89 | | 3.92 | | | 3.89 | | | 3.72 | | | 3.57 | | | 3.81 | | | 3.68 | | | 3.56 | | | 3.40 | | 3.54 | | 3.54 | | |  |  |
|  | GE | **2.85** | | **2.88** | | | **2.86** | | | **2.74** | | | **2.64** | | | **2.76** | | | **2.73** | | | **2.66** | | | **2.52** | | **2.63** | | **2.63** | | | \|error\| ≤ 2.0 |  |
| WS (ms^-1^) | OBS | 3.47 | | 3.44 | | | 3.41 | | | 3.41 | | | 3.41 | | | 3.37 | | | 3.35 | | | 3.32 | | | 3.36 | | 3.37 | | 3.39 | | |  |  |
|  | PRE | 4.21 | | 4.14 | | | 4.17 | | | 4.21 | | | 4.20 | | | 4.22 | | | 4.21 | | | 4.16 | | | 4.21 | | 4.27 | | 4.34 | | |  |  |
|  | MB | **0.75** | | **0.71** | | | **0.76** | | | **0.79** | | | **0.79** | | | **0.84** | | | **0.86** | | | **0.84** | | | **0.85** | | **0.90** | | **0.95** | | | \|error\| ≤ 0.5 |  |
|  | RMSE | 2.00 | | 1.97 | | | 1.98 | | | **2.02** | | | 2.00 | | | **2.03** | | | **2.03** | | | **2.01** | | | **2.03** | | **2.09** | | **2.12** | | | \|error\| ≤ 2.0 |  |
|  | GE | 1.54 | | 1.51 | | | 1.52 | | | 1.54 | | | 1.54 | | | 1.56 | | | 1.56 | | | 1.54 | | | 1.56 | | 1.61 | | 1.64 | | | \|error\| ≤ 2.0 |  |
| WD (°) | OBS | 175.84 | 174.28 | | | 175.20 | | | 176.02 | | | 175.68 | | | 176.16 | | | 174.87 | | | 173.35 | | | 175.23 | | | 173.25 | | | 172.67 | |  |  |
|  | PRE | 175.94 | 175.23 | | | 175.78 | | | 178.91 | | | 178.80 | | | 179.71 | | | 176.31 | | | 174.30 | | | 178.25 | | | 176.35 | | | 176.13 | |  |  |
|  | MB | 5.93 | | 6.67 | | | 6.27 | | | 8.21 | | | 7.81 | | | 8.30 | | | 7.06 | | | 6.62 | | | 7.72 | | 8.36 | | 9.22 | | | \|error\| ≤ 10 |  |
|  | RMSE | 62.68 | | 62.33 | | | 62.41 | | | 61.79 | | | 62.04 | | | 60.64 | | | 60.96 | | | 60.90 | | | 60.46 | | 60.39 | | 60.63 | | |  |  |
|  | GE | **46.15** | | **45.75** | | | **45.71** | | | **45.24** | | | **45.13** | | | **43.91** | | | **44.23** | | | **44.20** | | | **43.79** | | **43.83** | | **43.97** | | | \|error\| ≤ 30 |  |
| RH (%) | OBS | 73.60 | | 72.05 | | | 71.47 | | | 72.16 | | | 70.78 | | | 70.91 | | | 71.23 | | | 70.57 | | | 70.80 | | 70.11 | | 71.11 | | |  |  |
|  | PRE | 73.31 | | 68.66 | | | 70.76 | | | 69.49 | | | 71.04 | | | 71.13 | | | 70.02 | | | 69.20 | | | 71.52 | | 70.84 | | 71.58 | | |  |  |
|  | MB | -0.29 | | -3.39 | | | -0.71 | | | -2.66 | | | 0.26 | | | 0.22 | | | -1.22 | | | -1.36 | | | 0.71 | | 0.73 | | 0.47 | | |  |  |
|  | RMSE | 15.40 | | 15.93 | | | 15.72 | | | 16.01 | | | 15.39 | | | 16.27 | | | 16.54 | | | 16.59 | | | 16.10 | | 16.50 | | 15.79 | | |  |  |
|  | GE | 11.82 | | 12.15 | | | 12.01 | | | 12.28 | | | 11.93 | | | 12.74 | | | 12.92 | | | 12.97 | | | 12.61 | | 12.90 | | 12.25 | | |  |  |
|  |  | 2011 | | | 2012 | | | 2013 | | | 2014 | | | 2015 | | | 2016 | | | 2017 | | | 2018 | | | 2019 | | 2020 | | |  |  | |
| T2 (K) | OBS | 285.46 | | | 287.49 | | | 287.33 | | | 286.22 | | | 287.47 | | | 286.39 | | | 286.55 | | | 286.35 | | | 286.61 | | 286.74 | | |  |  | |
|  | PRE | 284.68 | | | 286.88 | | | 286.63 | | | 285.66 | | | 286.89 | | | 286.08 | | | 285.86 | | | 285.77 | | | 285.91 | | 286.62 | | |  |  | |
|  | MB | **-0.76** | | | **-0.59** | | | **-0.67** | | | **-0.53** | | | **-0.56** | | | -0.45 | | | **-0.67** | | | **-0.55** | | | **-0.67** | | -0.11 | | | \|error\| ≤ 0.5 | | |
|  | RMSE | 3.61 | | | 3.48 | | | 3.61 | | | 3.55 | | | 3.46 | | | 3.42 | | | 3.47 | | | 3.46 | | | 3.53 | | 3.69 | | |  |  | |
|  | GE | **2.69** | | | **2.58** | | | **2.68** | | | **2.62** | | | **2.56** | | | **2.58** | | | **2.56** | | | **2.54** | | | **2.58** | | **2.62** | | | \|error\| ≤ 2.0 | | |
| WS (ms^-1^) | OBS | 3.36 | | | 3.36 | | | 3.38 | | | 3.31 | | | 3.36 | | | 3.44 | | | 3.36 | | | 3.38 | | | 3.32 | | 3.14 | | |  |  | |
|  | PRE | 4.23 | | | 4.21 | | | 4.31 | | | 4.12 | | | 4.16 | | | 3.89 | | | 4.16 | | | 4.22 | | | 4.20 | | 3.94 | | |  |  | |
|  | MB | **0.86** | | | **0.85** | | | **0.92** | | | **0.81** | | | **0.81** | | | **0.81** | | | **0.80** | | | **0.84** | | | **0.88** | | **0.79** | | | \|error\| ≤ 0.5 | | |
|  | RMSE | **2.06** | | | **2.05** | | | **2.10** | | | **2.02** | | | **2.03** | | | **2.01** | | | **2.04** | | | **2.06** | | | **2.08** | | **2.39** | | | \|error\| ≤ 2.0 | | |
|  | GE | 1.58 | | | 1.58 | | | 1.62 | | | 1.55 | | | 1.56 | | | 1.57 | | | 1.56 | | | 1.58 | | | 1.60 | | 1.85 | | | \|error\| ≤ 2.0 | | |
| WD (°) | OBS | 173.85 | | | 173.00 | | | 174.57 | | | 173.22 | | | 172.35 | | | 174.50 | | | 176.31 | | | 172.58 | | | 174.32 | | 175.37 | | |  |  | |
|  | PRE | 173.62 | | | 175.29 | | | 176.64 | | | 174.60 | | | 174.12 | | | 160.78 | | | 177.41 | | | 173.60 | | | 174.16 | | 167.41 | | |  |  | |
|  | MB | 8.12 | | | 7.09 | | | 7.34 | | | 7.05 | | | 7.25 | | | 6.14 | | | 6.44 | | | 1.02 | | | 5.63 | | -0.73 | | | \|error\| ≤ 1.0 | | |
|  | RMSE | 60.78 | | | 60.75 | | | 60.10 | | | 61.33 | | | 61.03 | | | 59.51 | | | 61.10 | | | 60.35 | | | 61.75 | | 59.34 | | |  |  | |
|  | GE | **44.05** | | | **44.07** | | | **43.43** | | | **44.58** | | | **44.24** | | | **44.02** | | | **44.25** | | | **43.59** | | | **44.83** | | **45.13** | | | \|error\| ≤ 30 | | |
| RH (%) | OBS | 69.89 | | | 71.96 | | | 70.24 | | | 71.52 | | | 72.67 | | | 73.09 | | | 71.97 | | | 7_2.5_3 | | | 71.92 | | 72.12 | | |  |  | |
|  | PRE | 69.21 | | | 72.85 | | | 69.92 | | | 70.30 | | | 72.17 | | | 67.75 | | | 71.48 | | | 71.68 | | | 70.66 | | 75.05 | | |  |  | |
|  | MB | -0.63 | | | 0.90 | | | -0.32 | | | -1.22 | | | -0.50 | | | 0.27 | | | -0.49 | | | -0.85 | | | -1.26 | | 2.93 | | |  |  | |
|  | RMSE | 16.16 | | | 15.30 | | | 15.67 | | | 15.68 | | | 14.84 | | | 14.48 | | | 15.42 | | | 15.33 | | | 15.74 | | 18.77 | | |  |  | |
|  | GE | 12.59 | | | 11.81 | | | 12.11 | | | 12.06 | | | 11.39 | | | 11.24 | | | 11.84 | | | 11.74 | | | 12.06 | | 14.73 | | |  |  | |

**Table S7.** Model performance on PM_2.5_, in January to December from 2014 to 2020 (MFE is mean fractional error; MNB is mean normalized bias; MNE is mean normalized error). The criteria for PM_2.5_ are suggested by EPA[5].

| PM_2.5_ (μg·m^-3^) | |  | | Jan | Feb | Mar | Apr | May | Jun | Jul | Aug | Sep | Oct | Nov | Dec | | Criteria |
| --- | --- | --- | --- | --- | --- | --- | --- | --- | --- | --- | --- | --- | --- | --- | --- | --- | --- |
| 2014 | OBS | |  | |  |  |  | 59.14 | 52.08 | 51.16 | 43.23 | 43.51 | 69.83 | 73.79 | 74.70 |  | |
|  | PRE | |  | |  |  |  | 42.46 | 48.26 | 44.25 | 52.59 | 53.73 | 58.19 | 82.56 | 88.81 |  | |
|  | MNB | |  | |  |  |  | -0.15 | 0.14 | 0.06 | 0.39 | 0.43 | 0.07 | 0.42 | 0.46 |  | |
|  | MNE | |  | |  |  |  | 0.56 | 0.71 | 0.65 | 0.81 | 0.81 | 0.65 | 0.82 | 0.86 |  | |
|  | MFB | |  | |  |  |  | -0.41 | -0.21 | -0.24 | 0.00 | 0.04 | -0.23 | 0.02 | 0.04 | \|error\| ≤ 0.60 | |
|  | MFE | |  | |  |  |  | 0.64 | 0.61 | 0.61 | 0.59 | 0.58 | 0.61 | 0.60 | 0.61 | \|error\| ≤ 0.75 | |
| 2015 | OBS | | 85.17 | | 72.01 | 55.45 | 49.52 | 46.31 | 39.88 | 39.75 | 38.62 | 39.18 | 54.56 | 60.05 | 81.44 |  | |
|  | PRE | | 86.60 | | 72.98 | 52.39 | 38.76 | 32.84 | 31.47 | 33.99 | 42.74 | 44.87 | 48.93 | 72.60 | 89.80 |  | |
|  | MNB | | 0.21 | | 0.25 | 0.15 | -0.09 | -0.17 | -0.05 | 0.00 | 0.24 | 0.33 | 0.11 | 0.56 | 0.49 |  | |
|  | MNE | | 0.72 | | 0.76 | 0.73 | 0.60 | 0.59 | 0.65 | 0.64 | 0.75 | 0.81 | 0.68 | 0.99 | 0.93 |  | |
|  | MFB | | -0.13 | | -0.12 | -0.20 | -0.36 | -0.45 | -0.37 | -0.30 | -0.13 | -0.08 | -0.21 | 0.04 | 0.01 | \|error\| ≤ 0.60 | |
|  | MFE | | 0.62 | | 0.64 | 0.65 | 0.66 | 0.69 | 0.69 | 0.64 | 0.63 | 0.62 | 0.62 | 0.69 | 0.67 | \|error\| ≤ 0.75 | |
| 2016 | OBS | | 65.84 | | 60.44 | 61.32 | 45.13 | 41.42 | 34.62 | 33.89 | 32.14 | 40.84 | 41.33 | 63.17 | 81.65 |  | |
|  | PRE | | 61.56 | | 56.75 | 46.77 | 32.36 | 26.57 | 28.01 | 25.04 | 32.94 | 39.30 | 37.33 | 64.68 | 76.89 |  | |
|  | MNB | | 0.26 | | 0.17 | -0.08 | -0.16 | -0.25 | -0.08 | -0.17 | 0.14 | 0.07 | 0.12 | 0.29 | 0.15 |  | |
|  | MNE | | 0.76 | | 0.71 | 0.61 | 0.63 | 0.61 | 0.64 | 0.61 | 0.67 | 0.65 | 0.75 | 0.80 | 0.67 |  | |
|  | MFB | | -0.11 | | -0.17 | -0.36 | -0.48 | -0.56 | -0.40 | -0.47 | -0.17 | -0.23 | -0.27 | -0.11 | -0.16 | \|error\| ≤ 0.60 | |
|  | MFE | | 0.63 | | 0.65 | 0.66 | 0.74 | **0.76** | 0.70 | 0.71 | 0.60 | 0.62 | 0.68 | 0.66 | 0.61 | \|error\| ≤ 0.75 | |
| 2017 | OBS | | 74.96 | | 66.60 | 52.21 | 44.09 | 42.01 | 34.30 | 32.51 | 29.43 | 35.23 | 44.97 | 55.77 | 68.91 |  | |
|  | PRE | | 65.66 | | 56.80 | 43.38 | 31.38 | 25.60 | 26.83 | 23.42 | 26.65 | 32.79 | 37.95 | 52.62 | 67.08 |  | |
|  | MNB | | 0.12 | | 0.04 | 0.00 | -0.18 | -0.24 | -0.12 | -0.20 | 0.00 | 0.07 | 0.03 | 0.15 | 0.16 |  | |
|  | MNE | | 0.69 | | 0.64 | 0.67 | 0.61 | 0.59 | 0.64 | 0.57 | 0.67 | 0.68 | 0.66 | 0.70 | 0.69 |  | |
|  | MFB | | -0.21 | | -0.25 | -0.33 | -0.48 | -0.53 | -0.44 | -0.47 | -0.33 | -0.25 | -0.28 | -0.18 | -0.15 | \|error\| ≤ 0.60 | |
|  | MFE | | 0.64 | | 0.63 | 0.68 | 0.72 | 0.73 | 0.71 | 0.68 | 0.68 | 0.64 | 0.65 | 0.64 | 0.62 | \|error\| ≤ 0.75 | |
| 2018 | OBS | | 70.18 | | 59.84 | 53.49 | 45.12 | 37.21 | 31.41 | 28.54 | 27.91 | 27.90 | 37.75 | 53.38 | 70.18 |  | |
|  | PRE | | 63.47 | | 50.10 | 36.62 | 24.91 | 21.76 | 23.90 | 20.00 | 23.52 | 30.40 | 37.57 | 58.67 | 63.47 |  | |
|  | MNB | | 0.12 | | 0.02 | -0.17 | -0.34 | -0.33 | -0.15 | -0.22 | -0.08 | 0.19 | 0.14 | 0.32 | 0.12 |  | |
|  | MNE | | 0.71 | | 0.65 | 0.60 | 0.59 | 0.60 | 0.61 | 0.60 | 0.62 | 0.70 | 0.67 | 0.78 | 0.71 |  | |
|  | MFB | | -0.22 | | -0.28 | -0.46 | **-0.65** | **-0.63** | -0.45 | -0.51 | -0.37 | -0.14 | -0.17 | -0.05 | -0.22 | \|error\| ≤ 0.60 | |
|  | MFE | | 0.65 | | 0.66 | 0.71 | **0.80** | **0.79** | 0.69 | 0.72 | 0.66 | 0.60 | 0.60 | 0.62 | 0.65 | \|error\| ≤ 0.75 | |
| 2019 | OBS | | 71.54 | | 62.03 | 47.10 | 37.54 | 33.88 | 27.49 | 26.25 | 25.32 | 31.38 | 39.14 | 46.13 | 61.07 |  | |
|  | PRE | | 64.29 | | 51.09 | 38.59 | 28.17 | 25.91 | 29.97 | 26.96 | 32.81 | 32.40 | 35.70 | 46.87 | 64.50 |  | |
|  | MNB | | 0.15 | | 0.08 | -0.01 | -0.13 | -0.11 | 0.22 | 0.15 | 0.38 | 0.11 | 0.07 | 0.19 | 0.27 |  | |
|  | MNE | | 0.73 | | 0.69 | 0.64 | 0.59 | 0.57 | 0.73 | 0.67 | 0.82 | 0.60 | 0.63 | 0.69 | 0.73 |  | |
|  | MFB | | -0.21 | | -0.25 | -0.31 | -0.40 | -0.37 | -0.13 | -0.17 | -0.01 | -0.15 | -0.21 | -0.12 | -0.07 | \|error\| ≤ 0.60 | |
|  | MFE | | 0.65 | | 0.65 | 0.66 | 0.67 | 0.64 | 0.60 | 0.61 | 0.64 | 0.56 | 0.59 | 0.60 | 0.61 | \|error\| ≤ 0.75 | |
| 2020 | OBS | | 71.38 | | 46.11 | 38.16 | 38.68 | 30.30 | 26.60 | 25.93 | 24.19 | 27.57 | 36.11 | 43.64 | 60.99 |  | |
|  | PRE | | 78.48 | | 65.75 | 39.49 | 30.77 | 22.63 | 21.66 | 23.09 | 27.98 | 35.98 | 34.46 | 46.36 | 71.89 |  | |
|  | MNB | | 0.41 | | 0.75 | 0.21 | -0.05 | -0.15 | -0.07 | -0.01 | 0.24 | 0.41 | 0.13 | 0.26 | 0.36 |  | |
|  | MNE | | 0.85 | | 1.10 | 0.72 | 0.61 | 0.56 | 0.64 | 0.64 | 0.77 | 0.85 | 0.71 | 0.75 | 0.76 |  | |
|  | MFB | | -0.02 | | 0.15 | -0.13 | -0.33 | -0.41 | -0.38 | -0.32 | -0.14 | -0.02 | -0.22 | -0.10 | 0.01 | \|error\| ≤ 0.60 | |
|  | MFE | | 0.63 | | 0.66 | 0.60 | 0.64 | 0.65 | 0.67 | 0.65 | 0.65 | 0.62 | 0.62 | 0.60 | 0.58 | \|error\| ≤ 0.75 | |

**Table S8.** Model performance on PM_2.5_ from 2014 to 2020 in different regions.

| PM_2.5_ (μg·m^-3^) |  | China | BTH | YRD | PRD | SCB | FWP | Criteria |
| --- | --- | --- | --- | --- | --- | --- | --- | --- |
| 2014 | OBS | 58.84 | 86.00 | 59.44 | 40.06 | 57.98 | 58.97 |  |
|  | PRE | 60.57 | 79.65 | 58.80 | 24.73 | 122.07 | 66.25 |  |
|  | MNB | 0.26 | 0.27 | 0.11 | -0.31 | 1.73 | 0.49 |  |
|  | MNE | 0.75 | 0.74 | 0.63 | 0.46 | 1.84 | 0.87 |  |
|  | MFB | -0.10 | -0.08 | -0.16 | -0.49 | 0.58 | 0.07 | \|error\| ≤ 0.60 |
|  | MFE | 0.60 | 0.59 | 0.57 | 0.59 | 0.73 | 0.59 | \|error\| ≤ 0.75 |
| 2015 | OBS | 55.15 | 80.80 | 56.09 | 36.46 | 57.40 | 61.80 |  |
|  | PRE | 53.98 | 70.61 | 58.67 | 24.90 | 97.18 | 63.35 |  |
|  | MNB | 0.17 | 0.15 | 0.17 | -0.21 | 1.13 | 0.37 |  |
|  | MNE | 0.74 | 0.69 | 0.66 | 0.47 | 1.35 | 0.78 |  |
|  | MFB | -0.19 | -0.16 | -0.12 | -0.39 | 0.36 | -0.01 | \|error\| ≤ 0.60 |
|  | MFE | 0.65 | 0.61 | 0.57 | 0.56 | 0.66 | 0.58 | \|error\| ≤ 0.75 |
| 2016 | OBS | 50.15 | 72.06 | 48.71 | 34.25 | 54.35 | 66.82 |  |
|  | PRE | 44.29 | 59.54 | 46.33 | 20.21 | 85.05 | 53.81 |  |
|  | MNB | 0.03 | 0.09 | 0.05 | -0.34 | 0.89 | 0.08 |  |
|  | MNE | 0.68 | 0.65 | 0.61 | 0.50 | 1.16 | 0.62 |  |
|  | MFB | -0.29 | -0.20 | -0.22 | -0.56 | 0.26 | -0.19 | \|error\| ≤ 0.60 |
|  | MFE | 0.67 | 0.61 | 0.59 | 0.67 | 0.62 | 0.57 | \|error\| ≤ 0.75 |
| 2017 | OBS | 48.61 | 64.73 | 45.63 | 36.22 | 49.65 | 67.16 |  |
|  | PRE | 41.00 | 51.69 | 43.45 | 19.86 | 76.82 | 46.35 |  |
|  | MNB | -0.02 | -0.01 | 0.04 | -0.38 | 0.97 | -0.12 |  |
|  | MNE | 0.65 | 0.59 | 0.60 | 0.51 | 1.21 | 0.54 |  |
|  | MFB | -0.32 | -0.27 | -0.22 | **-0.61** | 0.31 | -0.34 | \|error\| ≤ 0.60 |
|  | MFE | 0.67 | 0.60 | 0.59 | 0.70 | 0.64 | 0.59 | \|error\| ≤ 0.75 |
| 2018 | OBS | 44.52 | 59.85 | 44.30 | 32.99 | 43.47 | 59.84 |  |
|  | PRE | 40.27 | 48.51 | 40.26 | 17.48 | 67.26 | 40.92 |  |
|  | MNB | -0.03 | 0.04 | -0.03 | -0.41 | 0.89 | -0.13 |  |
|  | MNE | 0.65 | 0.61 | 0.60 | 0.53 | 1.14 | 0.55 |  |
|  | MFB | -0.35 | -0.23 | -0.28 | **-0.65** | 0.29 | -0.36 | \|error\| ≤ 0.60 |
|  | MFE | 0.68 | 0.59 | 0.61 | 0.74 | 0.63 | 0.61 | \|error\| ≤ 0.75 |
| 2019 | OBS | 43.05 | 53.69 | 41.37 | 31.53 | 40.45 | 57.04 |  |
|  | PRE | 40.15 | 46.11 | 42.10 | 21.93 | 74.09 | 42.02 |  |
|  | MNB | 0.11 | 0.08 | 0.15 | -0.20 | 1.19 | -0.01 |  |
|  | MNE | 0.67 | 0.62 | 0.65 | 0.46 | 1.37 | 0.54 |  |
|  | MFB | -0.21 | -0.19 | -0.13 | -0.37 | 0.44 | -0.24 | \|error\| ≤ 0.60 |
|  | MFE | 0.62 | 0.59 | 0.58 | 0.54 | 0.67 | 0.56 | \|error\| ≤ 0.75 |
| 2020 | OBS | 39.79 | 48.75 | 35.39 | 26.93 | 37.02 | 50.45 |  |
|  | PRE | 39.29 | 46.81 | 46.87 | 25.96 | 74.57 | 42.24 |  |
|  | MNB | 0.21 | 0.11 | 0.44 | 0.07 | 1.39 | 0.02 |  |
|  | MNE | 0.75 | 0.69 | 0.86 | 0.53 | 1.58 | 0.57 |  |
|  | MFB | -0.15 | -0.21 | 0.00 | -0.13 | 0.45 | -0.22 | \|error\| ≤ 0.60 |
|  | MFE | 0.63 | 0.62 | 0.60 | 0.50 | 0.72 | 0.56 | \|error\| ≤ 0.75 |

**Table S9.** Verification of CMAQ simulation results for urban and non-urban areas

| **Year** | **Indicator** | **Urban** | **Non-urban** | **Criteria** |
| --- | --- | --- | --- | --- |
| 2014 | OBS | 59.54 | 55.03 |  |
|  | PRE | 59.84 | 58.66 |  |
|  | MNB | 0.21 | 0.32 |  |
|  | MNE | 0.71 | 0.81 |  |
|  | MFB | -0.12 | -0.08 | \|error\| ≤ 0.60 |
|  | MFE | 0.60 | 0.62 | \|error\| ≤ 0.75 |
| 2015 | OBS | 56.20 | 51.09 |  |
|  | PRE | 55.44 | 51.85 |  |
|  | MNB | 0.17 | 0.22 |  |
|  | MNE | 0.72 | 0.79 |  |
|  | MFB | -0.17 | -0.17 | \|error\| ≤ 0.60 |
|  | MFE | 0.63 | 0.67 | \|error\| ≤ 0.75 |
| 2016 | OBS | 50.92 | 47.67 |  |
|  | PRE | 45.98 | 43.67 |  |
|  | MNB | 0.05 | 0.08 |  |
|  | MNE | 0.66 | 0.71 |  |
|  | MFB | -0.26 | -0.27 | \|error\| ≤ 0.60 |
|  | MFE | 0.65 | 0.67 | \|error\| ≤ 0.75 |
| 2017 | OBS | 49.50 | 46.10 |  |
|  | PRE | 44.18 | 42.63 |  |
|  | MNB | -0.01 | 0.04 |  |
|  | MNE | 0.63 | 0.68 |  |
|  | MFB | -0.31 | -0.29 | \|error\| ≤ 0.60 |
|  | MFE | 0.65 | 0.67 | \|error\| ≤ 0.75 |
| 2018 | OBS | 45.05 | 42.15 |  |
|  | PRE | 42.31 | 41.67 |  |
|  | MNB | -0.03 | 0.02 |  |
|  | MNE | 0.64 | 0.69 |  |
|  | MFB | -0.33 | -0.32 | \|error\| ≤ 0.60 |
|  | MFE | 0.66 | 0.68 | \|error\| ≤ 0.75 |
| 2019 | OBS | 43.88 | 40.62 |  |
|  | PRE | 40.92 | 40.24 |  |
|  | MNB | 0.09 | 0.18 |  |
|  | MNE | 0.65 | 0.72 |  |
|  | MFB | -0.20 | -0.09 | \|error\| ≤ 0.60 |
|  | MFE | 0.60 | 0.63 | \|error\| ≤ 0.75 |
| 2020 | OBS | 40.21 | 37.43 |  |
|  | PRE | 40.64 | 39.63 |  |
|  | MNB | 0.26 | 0.34 |  |
|  | MNE | 0.74 | 0.82 |  |
|  | MFB | -0.09 | -0.07 | \|error\| ≤ 0.60 |
|  | MFE | 0.60 | 0.63 | \|error\| ≤ 0.75 |

**Table S10.** Comparison of results from previous source resolution studies with our simulation results

| Time | City | Previous Studies | Method | This Study | Ref. |
| --- | --- | --- | --- | --- | --- |
| 2012 Jul | Beijing | Power: 8%  Residential: 21%  Transportation: 12%  Agricultural: 6%  Industry: 33% | CAMx/PSAT | Power: 7.5%  Residential: 26.35%  Transportation: 5.28%  Agricultural: 13.02%  Industry: 41.75% | [6] |
| 2013 | BTH | Power: 8%  Industry: 44%;  Residential: 30%  Transportation: 7%  Agriculture: 8%  SOA: 1.2% | CAMX-PSAT | Power: 7.5%  Industry: 44.1%;  Residential: 23.78%  Transportation: 7.82%  Agriculture: 11.1%  SOA: 3.5% | [7] |
| 2013-2017 | Shanghai | Power:10%;  Residential: 25%;  Transportation: 13%;  Agricultural: 11%;  Industry: 40%;  Biogenic: 0.85% | CAMx/PSAT | Power:9.9%;  Residential: 13.5%;  Transportation: 4.5%;  Agricultural: 13.85%;  Industry: 48.1%;  Biogenic: 0.88% | [8] |
| 2020 | Chengdu | Power:1.1%;  Industry: 25%;  Residential: 27%;  Transportation: 6.8%;  Agriculture:5.2% | CMAQ-ISAM | Power:2.75%;  Industry: 31.99%;  Residential: 22.75%;  Transportation: 13.57%;  Agriculture:9.38% | [9] |
| 2017 | PRD | Dust:  29%-34% (RSM);  27%-31% (PSAT);  Mobile:  16%-25% (RSM);  19%-30% (PAST) | Response Surface Model (RSM) and PSAT | Transportation:8.5%;  Dust:3.7% | [10] |
| 2015 | Guangzhou | Mobile:12%;  Power:3%;  Industry:4% | CAMX-PSAT | Mobile:3.65%;  Power:7.0%;  Industry:41.33% | [11] |
| 2006 | Guangzhou | Mobile:34%;  Power:6%;  Industry:4% | CAMX-PSAT | Mobile:6.5%;  Power:9.6%;  Industry:50.76% | [11] |
| 2018-2019 | Beijing | Coal combustion: 13.26%;  Vehicle source: 16.1%;  Industry source: 6.81%;  Dust: 1.81% | CAMx/PSAT-PMF | Coal combustion: 5.3%;  Vehicle source:11.7%;  Industry source: 36.31%;  Dust:4.2% | [12] |
| 2015-2016 | Shanghai | Coal combustion: 5.6%;  Industry: 20.2%;  Vehicle emission: 18.3%;  Dust: 4.6% | PMF | Power:6.0%;  Industry:34.8%;  Transportation:10.3%;  Dust:2.4% | [13] |
| 2015 | Guangzhou | Combustion:17.2%;  Agriculture: 5.7%;  Mobile: 5.3%;  Dust: 31.85% | response surface modeling technique with differential method（RSM-DM） | Combustion:6.56%;  Agriculture:12.9%;  Mobile: 3.65%;  Dust: 2.6% | [14] |
| 2017.11-2018.01 | Shijiazhuang | Power: 2.8%;  Industry: 20.8%;  Vehicle: 27.7%;  Dust: 1.5% | PMF-CALPUFF | Power:7.1%;  Residential:36.8%;  Transportation:7.15%;  Dust:1.1% | [15] |
| 2014 | Jinan | coal combustion: 21%;  industry: 13%;  motor vehicle exhaust:12% | PMF | Power:8.6%;  Industry:38.2%;  Transportation:7.8%; | [16] |
| 2016.2.25-3.22 | Yulin | Fossil fuel combustion: 9%;  Industry: 9%;  Traffic: 20%;  Dust: 10% | PMF | Power:3.5%;  Industry:25.7%;  Transportation:5.0%;  Dust:2.1% | [17] |
| 2019 | Beijing | coal combustion: 6.42%;  soil source: 6.82% | dispersion normalized PMF (DN-PMF) | Power:3.6%;  Agriculture:7.2% | [18] |
| 2007-2008 Autumn | Tianjin | Coal combustion: 16%;  Vehicular emission: 34%  Soil dust: 10%;  Marine aerosol: 7%; | Receptor modeling | Power:7.8%;  Transportation:8.3%;  Dust:1.6%;  Sea salt: 0.04% | [19] |
| 2013  Jun-Nov | Tianjin | coal combustion:13.77%;  vehicle exhaust and road dust: 26.39% | Multilinear Engine 2-species ratios (ME2-SR) | Power:6.1%;  Transportation:5.5%; | [20] |
| 2014 Jul-August | Beijing | coal combustion: 15%-16%;  vehicle exhaust: 17%-22%;  crustal dust: 17%-22% | PMF | Power:6.8%;  Transportation:6.6%;  Dust:1.5% | [21] |
| 2014 | Zhoushan | Mobile: 8.96%;  Industry: 7.02% | CMAQ-CMB | Mobile: 4.34%;  Industry: 48.13% | [22] |
| 2013 | 25 cities in China | Power plants: 8.7%-12.7%;  Industry: 25.0%-38.6%;  Residential: 9.6%-27%;  Transportation: <8%;  Agriculture: 9.5%-12%;  SOA: 5.4%-15.5% | Source-Oriented CMAQ | Power:8.5%;  Industry:31.4%;  Residential:19.7%;  Transportation:5.6%;  Agriculture:12.1%;  SOA: 8.8% | [23] |
| 2015 | Chengdu | Power: 4.1%  Industry: 49.76%  Residential: 25.9%;  Transportation: 2.7%  Agriculture: 7.2%;  Biogenic: 5.2% | Source-Oriented CMAQ | Power: 2.75%  Industry: 42.37%  Residential: 23.47%;  Transportation: 9.61%  Agriculture: 13.22%;  Biogenic: 2.4% | [24] |
| 2018.05 | YRD | Power: 4.5-9%  Residential: 5.9%-12.2%  Transportation: 11.5%-16.2%  Agricultural: 9%-14.2%  Industry: 40%-50%;  Biogenic: 1.8% | Source-Oriented CMAQ | Power: 6.54  Residential: 18.78  Transportation: 15.57%  Agricultural: 11.94  Industry: 34.64;  Biogenic: 2.68 | [25] |
| 2016-2017 | Sichuan Basin | Residential: 68.1%;  Industry: 16.4 | WRF-Chem | Residential: 27.1%;  Industry: 34.1% | [26] |
| 2016 | China | Power:3%;  Industry:29%-51%;  Residential: 35%  Transportation:6%;  Agriculture:11%;  Nature: 12% | WRF-Chem | Power:6.9%;  Industry:31.9%;  Residential: 22.5  Transportation:9.5%;  Agriculture:11.3%;  Nature: 12.8% | [27] |
| 2007-2016 | North China | Fossil fuel: 10.9%;  Industrial: 30.5%;  Transportation: 7.3%;  Dust: 15.8%;  SOA: 9% | Review | Power:9.8%;  Industry:35.5%;  Transportation:4.9%;  Dust:8.6%;  SOA:4.75% | [28] |
|  | East China | Fossil fuel: 13.7%;  Industrial: 17.9%;  Transportation: 13.7%;  Dust: 19.3%;  SOA:11% |  | Power:9.8%;  Industry:37.6%;  Transportation:7.6%;  Dust:1.7%;  SOA: 6.8% |  |
|  | Central China | Fossil fuel: 18.6%;  Industrial: 28.4%;  Transportation: 16.3%;  Dust: 24%;  SOA: 8.75% |  | Power:9.5%;  Industry:35.4%;  Transportation:7.8%;  Dust:1.6%;  SOA: 5.9% |  |
|  | South China | Fossil fuel: 7.4%;  Industrial: 13.7%;  Transportation: 22.1%;  Dust: 6.3%;  SOA: 18.7% |  | Power:8.8%;  Industry:32.9%;  Transportation:4.3%;  Dust:2.1%;  SOA: 15.9% |  |
|  | Northeast China | Fossil fuel: 17.4%;  Industrial: 23.6%;  Transportation: 7.1%;  Dust: 20.5%;  SOA: 7.6% |  | Power:9.0%;  Industry:32.2%;  Transportation:6.1%;  Dust:3.4%:  SOA: 7.2% |  |
|  | Northwest China | Fossil fuel: 14.2%;  Industrial: 17.1%;  Transportation: 9.8%;  Dust: 30%;  SOA: 4.6% |  | Power:7.7%;  Industry:20.8%;  Transportation:3.8%;  Dust:32.5%;  SOA: 3.8% |  |
|  | Southwest China | Fossil fuel: 15.7%;  Industrial: 17.1%;  Transportation: 14.2%;  Dust: 22.8%;  SOA:13% |  | Power:8.6%;  Industry:30.0%;  Transportation:4.17%;  Dust:2.98%;  SOA:13.1% |  |

**Table S11.** Anthropogenic emission reduction measures taken by China during the two-phase air pollution prevention and control program (based on Zhang et al.**[29]** and Geng et al.**[30]**)

| **Source** | **Measures** | **Descriptions** | **Phase Ⅰ** | **Phase Ⅱ** |
| --- | --- | --- | --- | --- |
| **Power** | Phase out old power generation capacity | 9% and 92% equipped with de-S and de-N devices  71% meet “ultralow emission’ standard” | 25 GW of coal-fired power generation capacity were phased out | - |
| **Industry** | Strengthen industrial emission standards | Enforce ultra-low emission retrofits in power sector Enforce ultra-low emission retrofits in iron and steel  Tighten emission limit for cement industry | 770 GW  Emission limit in GB 4915-2013 | 170 GW  620 million ton  Special emission limit in GB 4915-2013 |
|  | Phase out small and polluting factories | "Small and polluting" enterprises were phased out or rectified | 62,000 enterprises | 360,000 enterprises |
|  | Phase out outdated industrial capacities | Phase out outdated coal-fired power generation Phase out outdated iron and steel capacity  Phase out outdated cement capacity  Phase out outdated flat glass capacity | 25 GW  200 million ton  250 million ton  110 million weight box | 20GW  120 million ton  140 million ton  50 million weight box |
|  | Upgrades on industrial boilers | Promote new emission standard  Phase out small boilers | GB 13271-2014  200 thousand | Ultra-low emission limit for large boilers  in key regions  110 thousand |
| **Residential** | Promote clean fuels in the residential sector | Upgraded to clean stoves  Applied washed clean coal  Coal substituted by natural gas and electricity | lower sulfur and ash  6 million households | 23 million households |
| **Transportation** | Control of mobile source emissions | Tighten emission standard for light duty vehicles Tighten emission standard for heavy duty vehicles Eliminate old and yellow label vehicles  Promote new energy vehicles  "Highway to Railway": Railway cargo volume  "Highway to Waterway": Waterway cargo volume | China 5  China 5  20 million  increased by 1.5 million decreased by 7% increased by 19% | China 6a  China 5  6 million  increased by 3.4 million increased by 23% increased by 14% |
| **Agriculture** | Management of agricultural source | Croplands applied with soil testing and formula fertilization technology  Reduce national fertilizer consumption | decreased by 1 % | 13.3 million ha  decreased by 10% |
| **ASOA** | Management of VOC emissions | Release VOC emission control standard  Release VOC content limit standard  Promote the use of water-based paints | 35% water-based paints in 2017 | GB 37823-2019, GB 37824-2019, GB 37822-2019  GB 18581-2020, GB 18582-2020, GB 33372-2020, GB 38508-2020  48% water-based paints in 2020 |

GB 4915-2013: Emission standard of air pollutants for cement industry

GB 13271-2014: Emission standard of air pollutants for boiler

GB 37823-2019: Emission standard of air pollutants for pharmaceutical industry

GB 37824-2019: Emission standard of air pollutants for paint, ink and adhesive industry GB 37822-2019: Standard for fugitive emission of volatile organic compounds

GB 18581-2020: Limit of harmful substances of woodware coatings

GB 18582-2020: Limit of harmful substances of architectural wall coatings

GB 33372-2020: Limit of volatile organic compounds content in adhesive

GB 38508-2020: Limits for volatile organic compounds content in cleaning agents


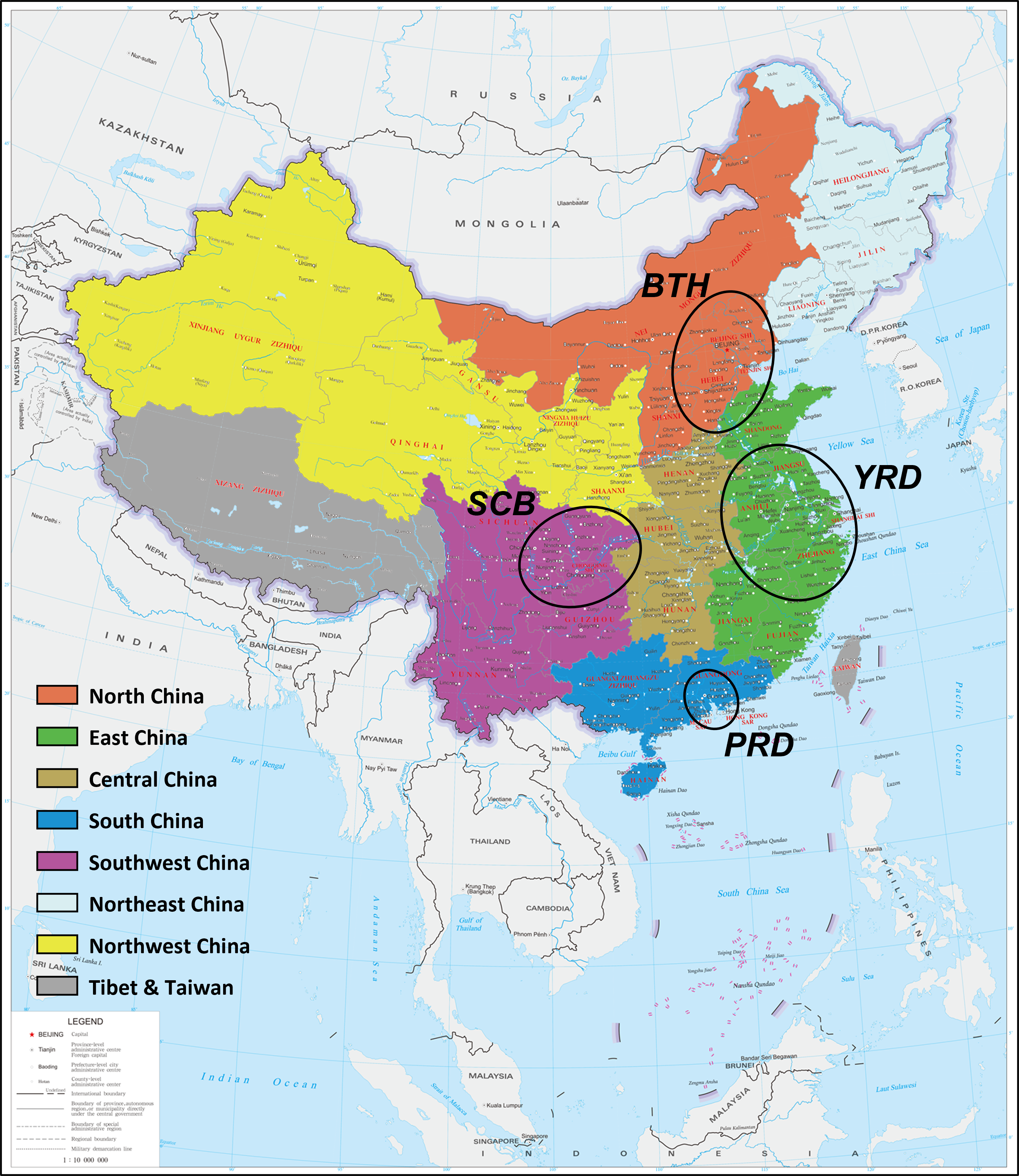


**Fig. S1.** Study domain and validation region divisions. The authority number of the base map: GS (2022) 4314. As Zhu et al. [28] excluded Tibet and Taiwan from their validation region divisions, these areas are consequently shown in gray on the map.


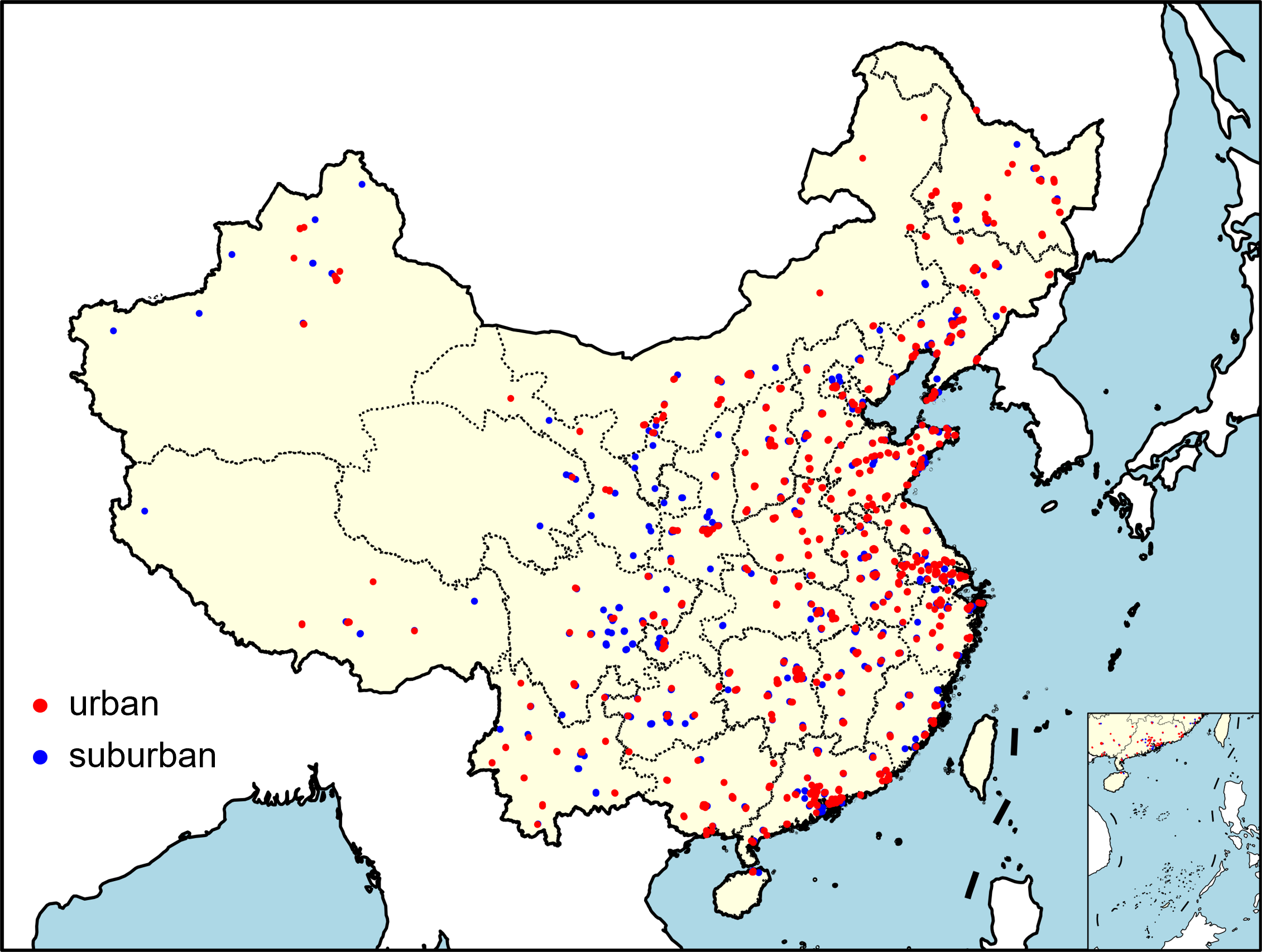


**Fig. S2.** Distribution of China National Environmental Monitoring Center (CNEMC) Monitoring Sites. The authority number of the base map: GS (2022) 4314.


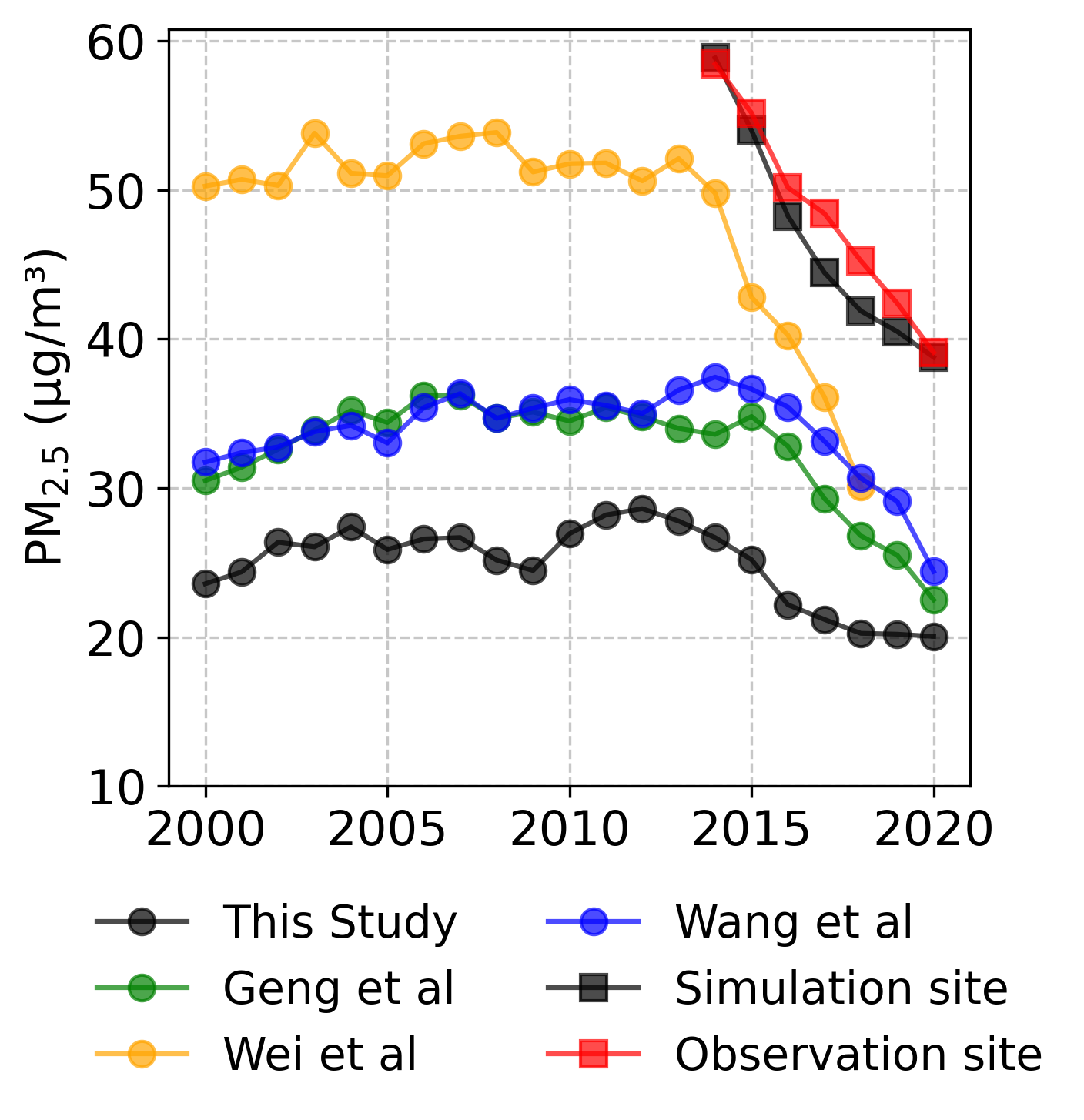


**Fig. S3.** Comparison of CMAQ simulation results with publicly available datasets (Nation-wide, 2000−2020) and validation of CMAQ site simulations with observational sites (2014−2020)


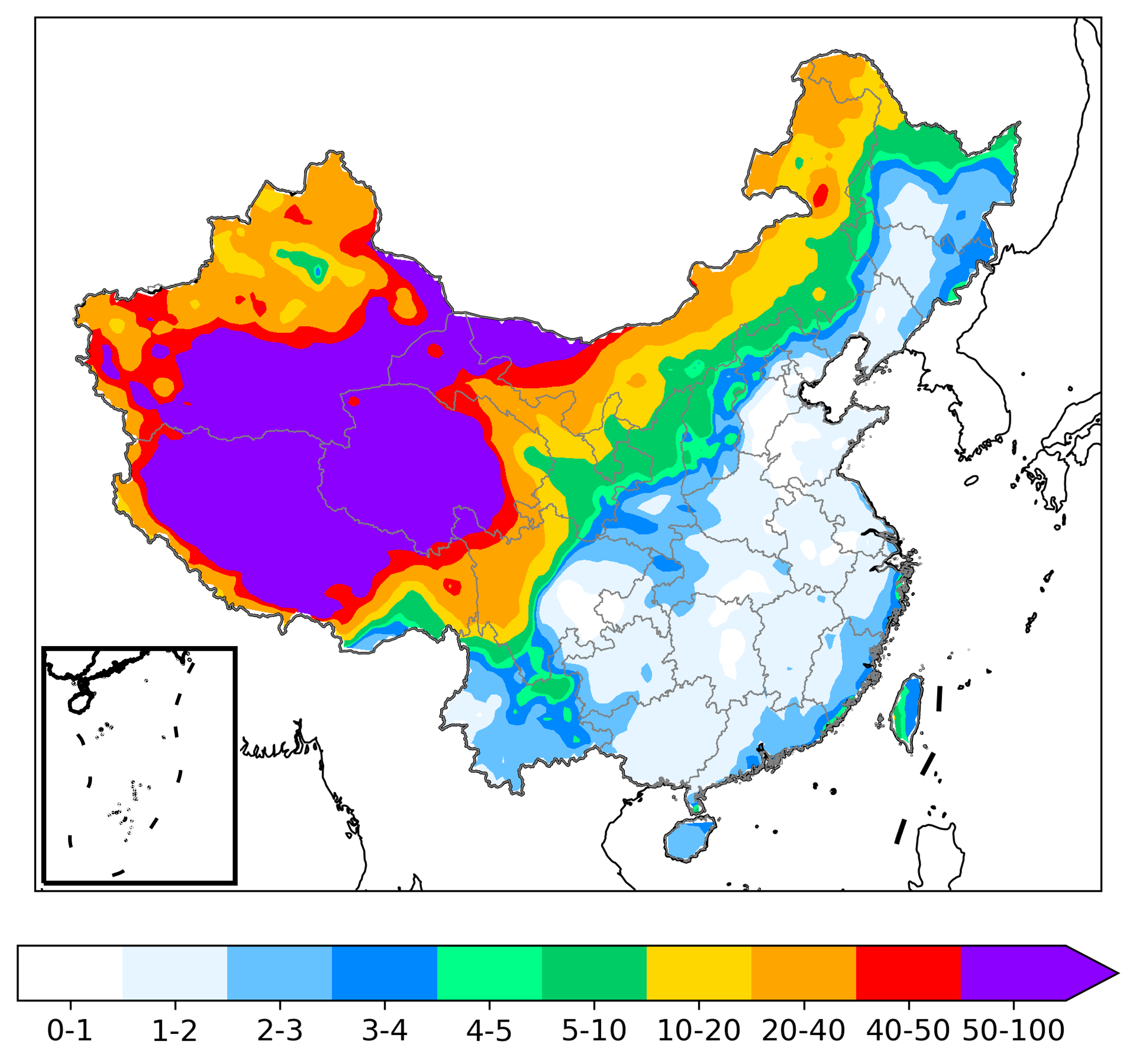


**Fig. S4.** Proportion of PM_2.5_ contributed by dust in the total concentration during 2000−2020. The authority number of the base map: GS (2022) 4314.


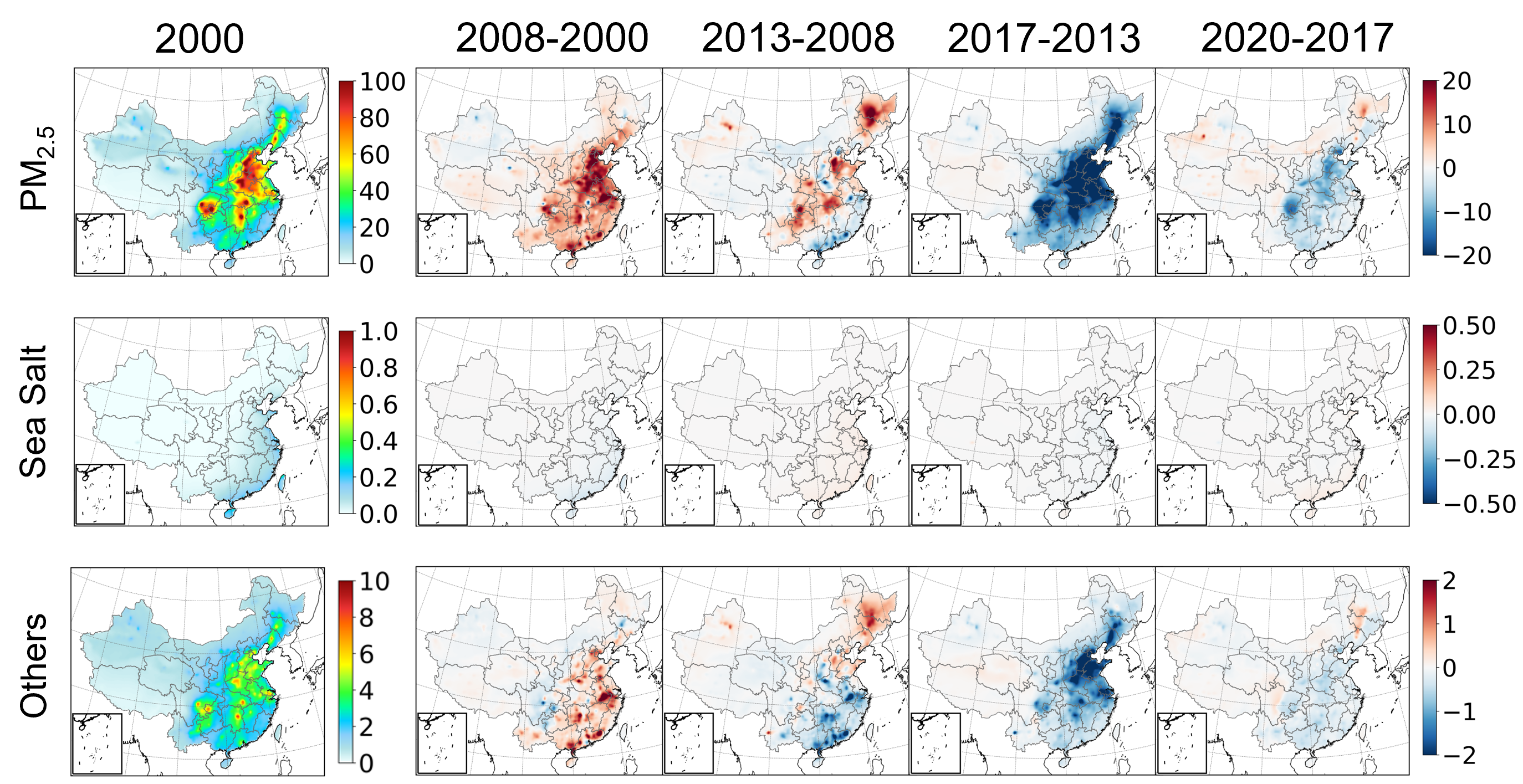


**Fig. S5.** Changes in the total PM_2.5_ concentrations and those contributed by sea salt and other sources across different periods (Units: µg/m³). The authority number of the base map: GS (2022) 4314.


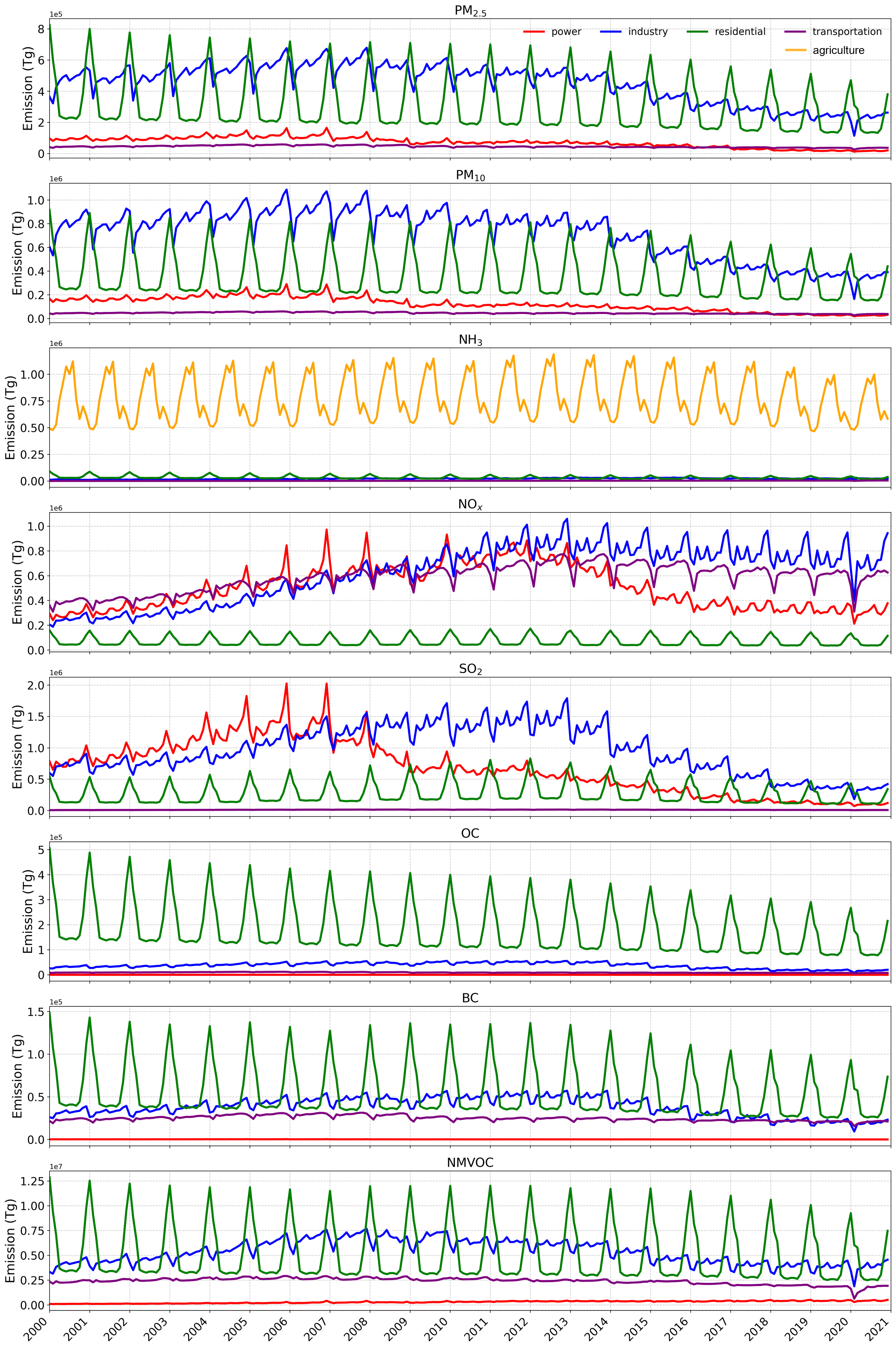


**Fig. S6. Changes in precursor emissions from different sources in China (2000–2020) based on MEICv1.4 (Unit: Tg)**


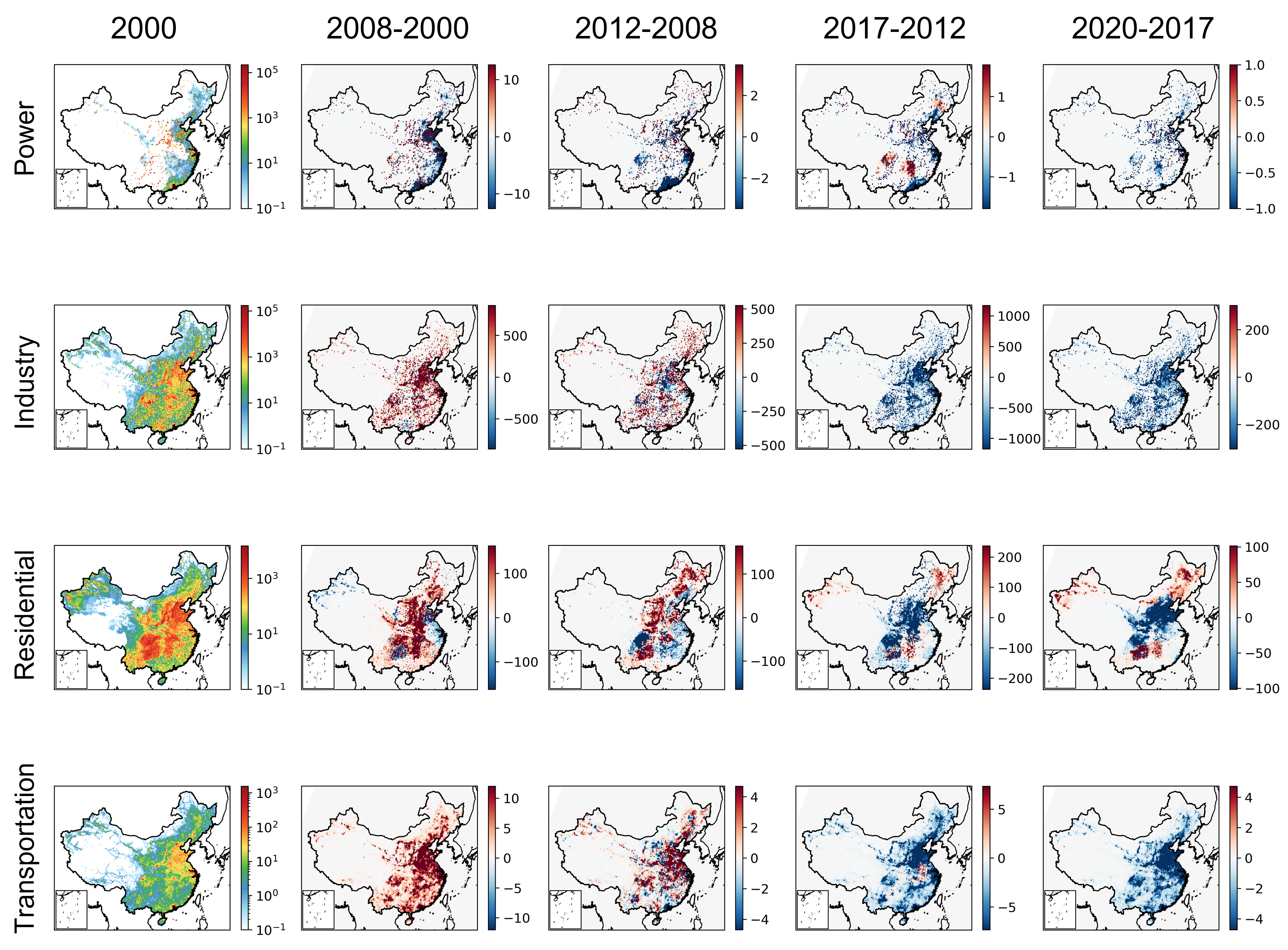


**Fig. S7.** Changes in anthropogenic NH_3_ emissions between different time periods (unit: Tg). The authority number of the base map: GS (2022) 4314.


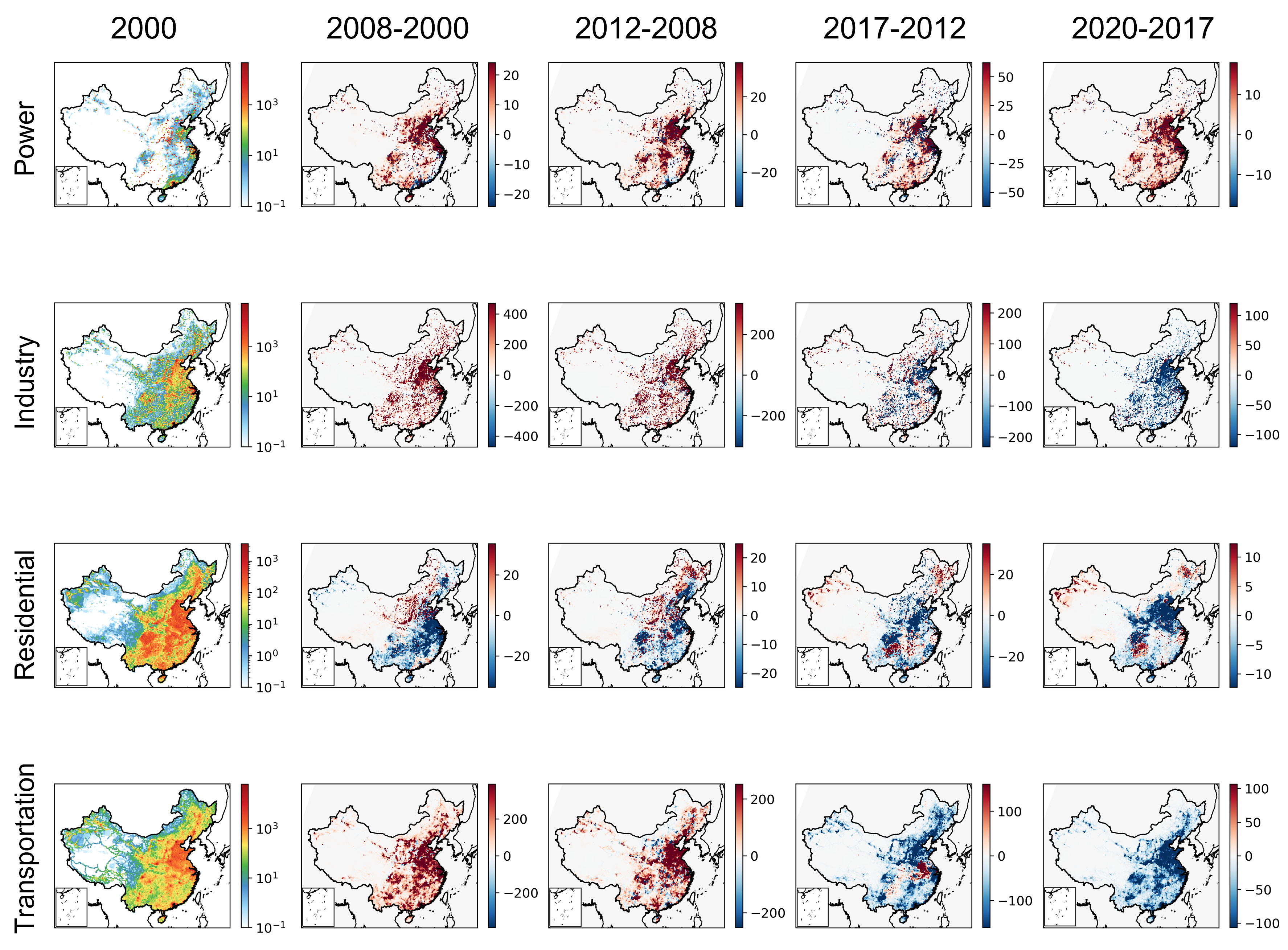


**Fig. S8.** Changes in anthropogenic NOx emissions between different time periods (unit: Tg). The authority number of the base map: GS (2022) 4314.


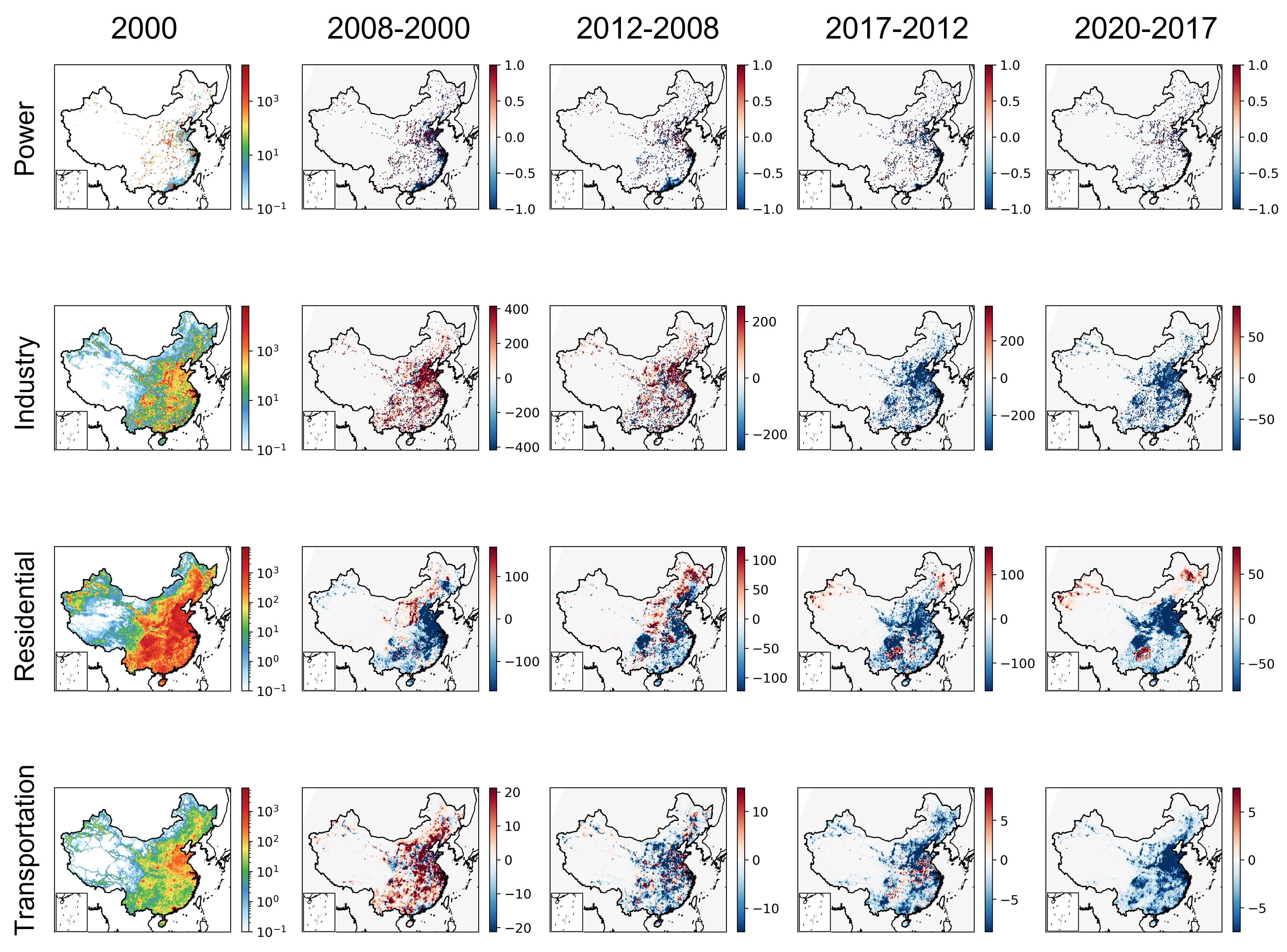


**Fig. S9.** Changes in anthropogenic PM_2.5_ emissions between different time periods (unit: Tg). The authority number of the base map: GS (2022) 4314.


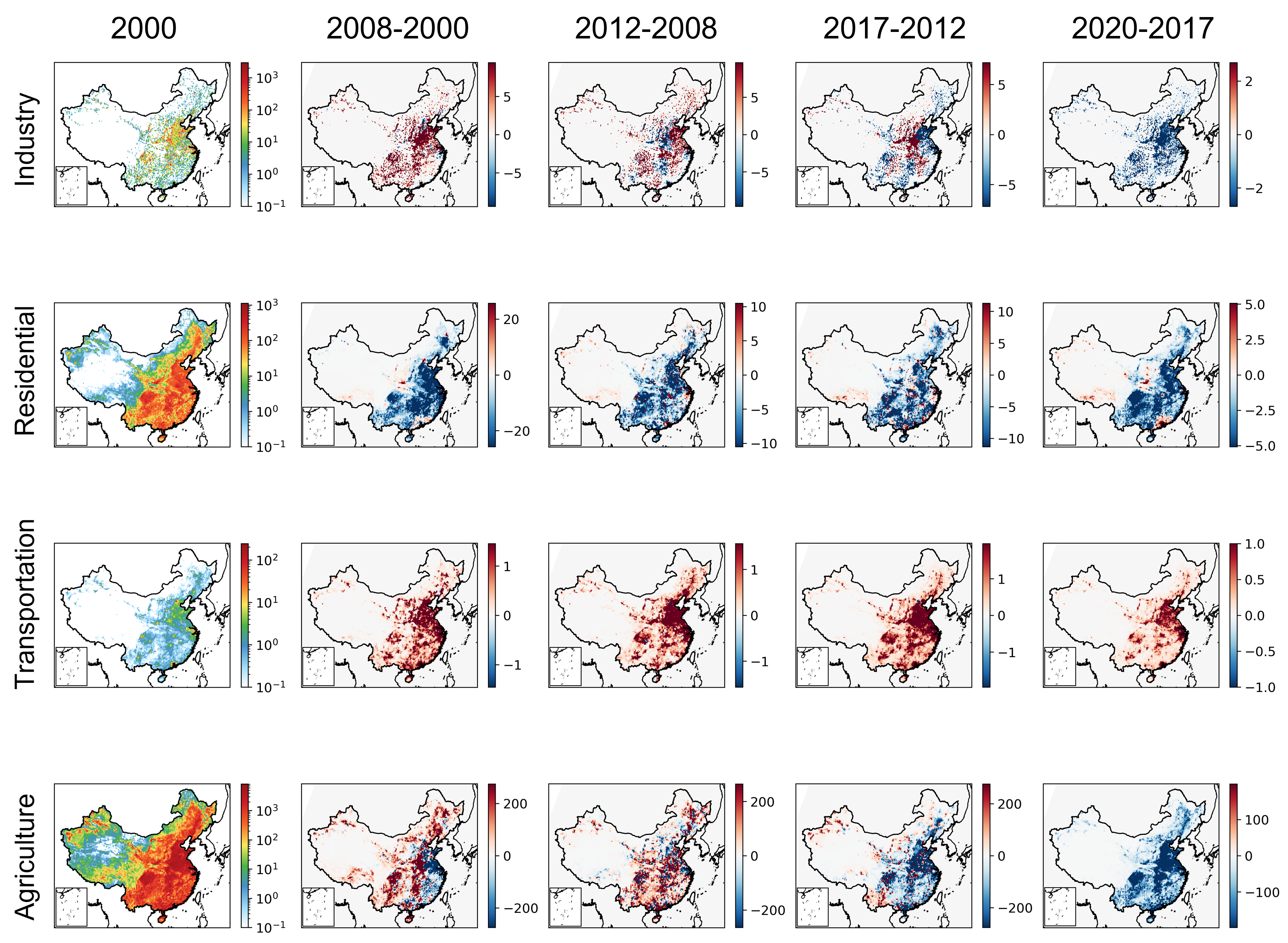


**Fig. S10.** Changes in anthropogenic SO_2_ emissions between different time periods (unit: Tg). The authority number of the base map: GS (2022) 4314.


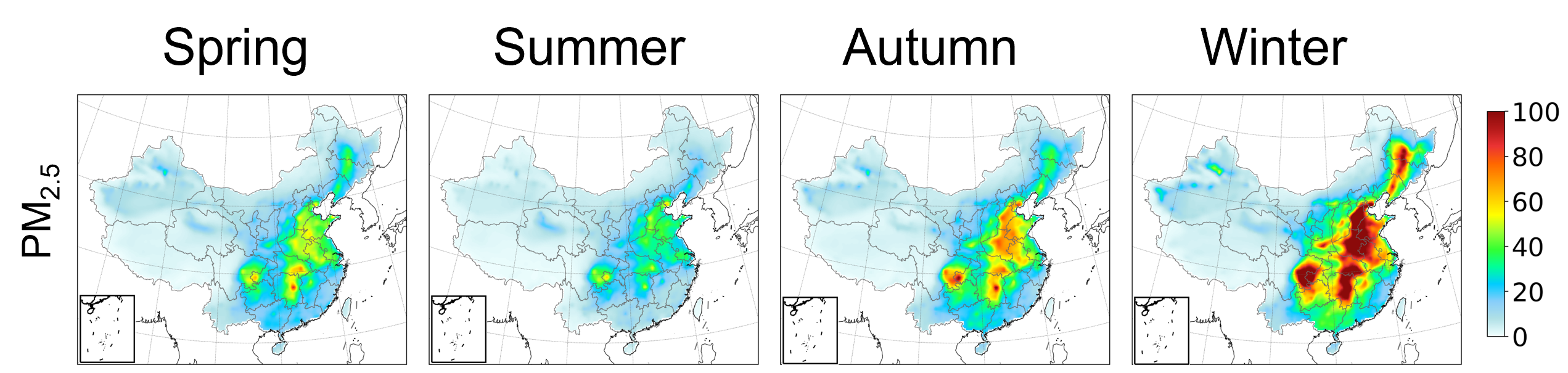


**Fig. S11.** Seasonal variations in PM_2.5_ concentrations during 2000−2020 (Units: µg/m³). The authority number of the base map: GS(2022)4314.


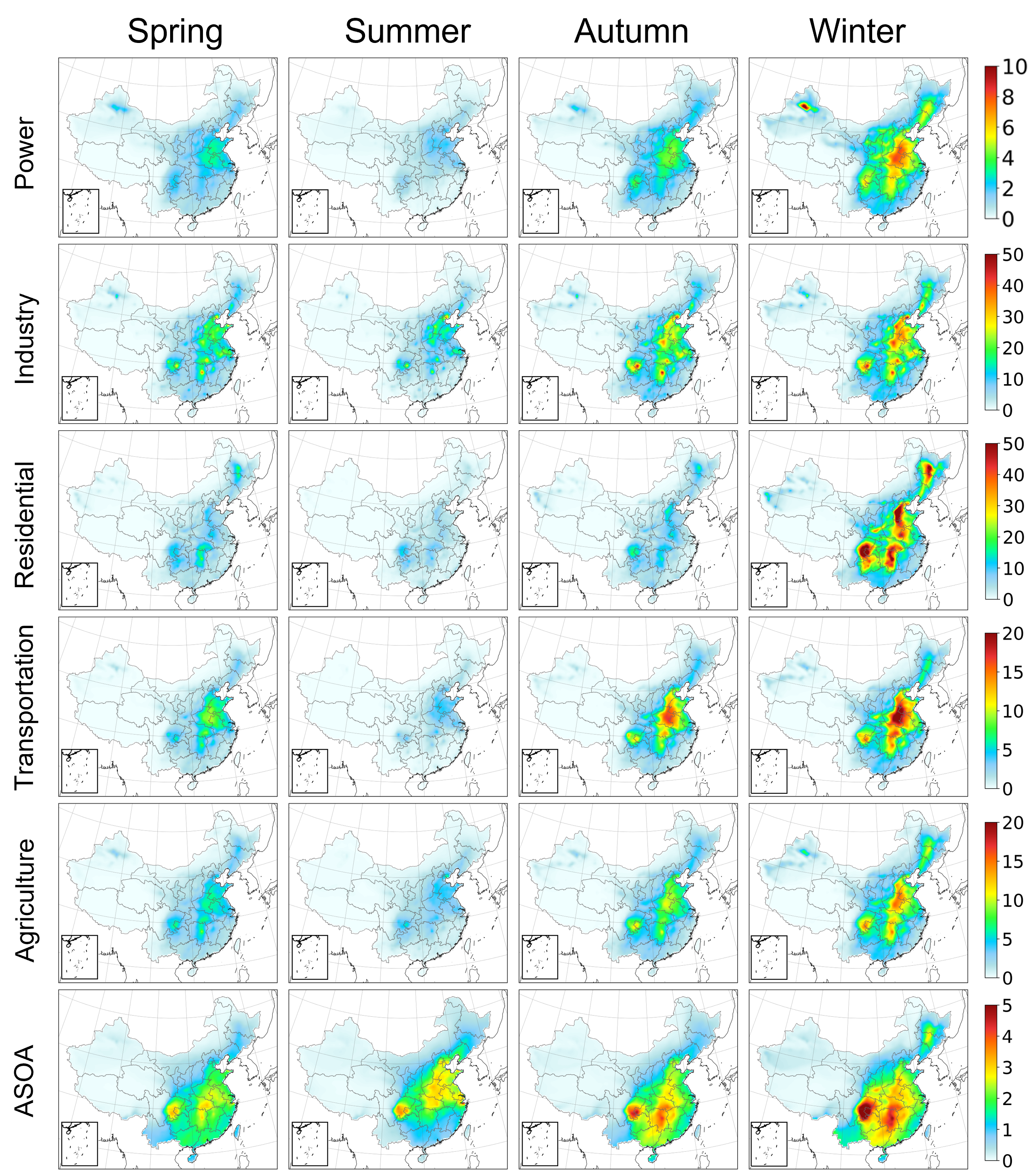


**Fig. S12.** Seasonal variations in PM_2.5_ concentrations contributed by anthropogenic sources during 2000-2020 (Units: µg/m³). The authority number of the base map: GS (2022) 4314.


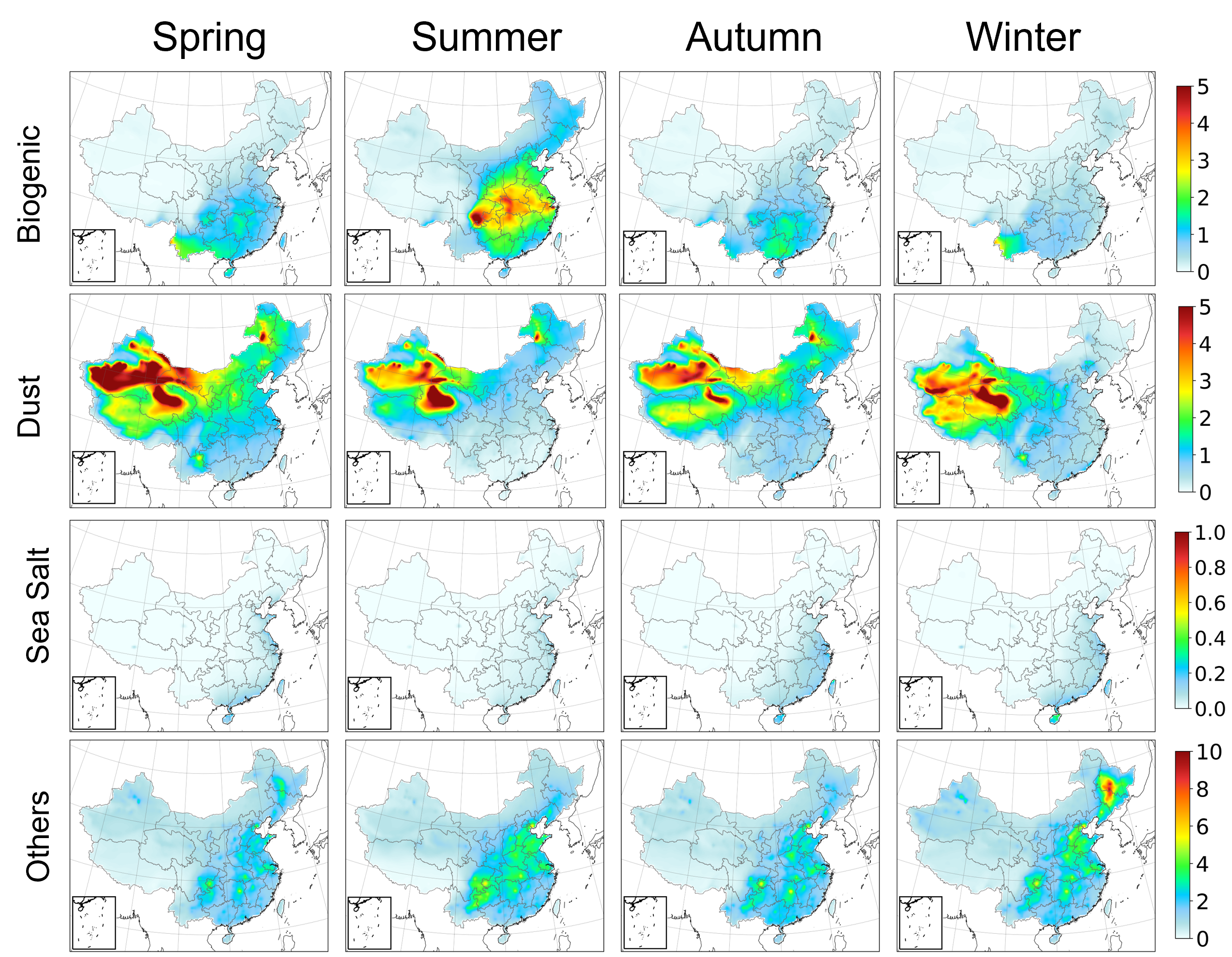


**Fig. S13.** Seasonal variations in PM_2.5_ concentrations contributed by nature and others sources during 2000−2020 (Units: µg/m³). The authority number of the base map: GS (2022) 4314.

**
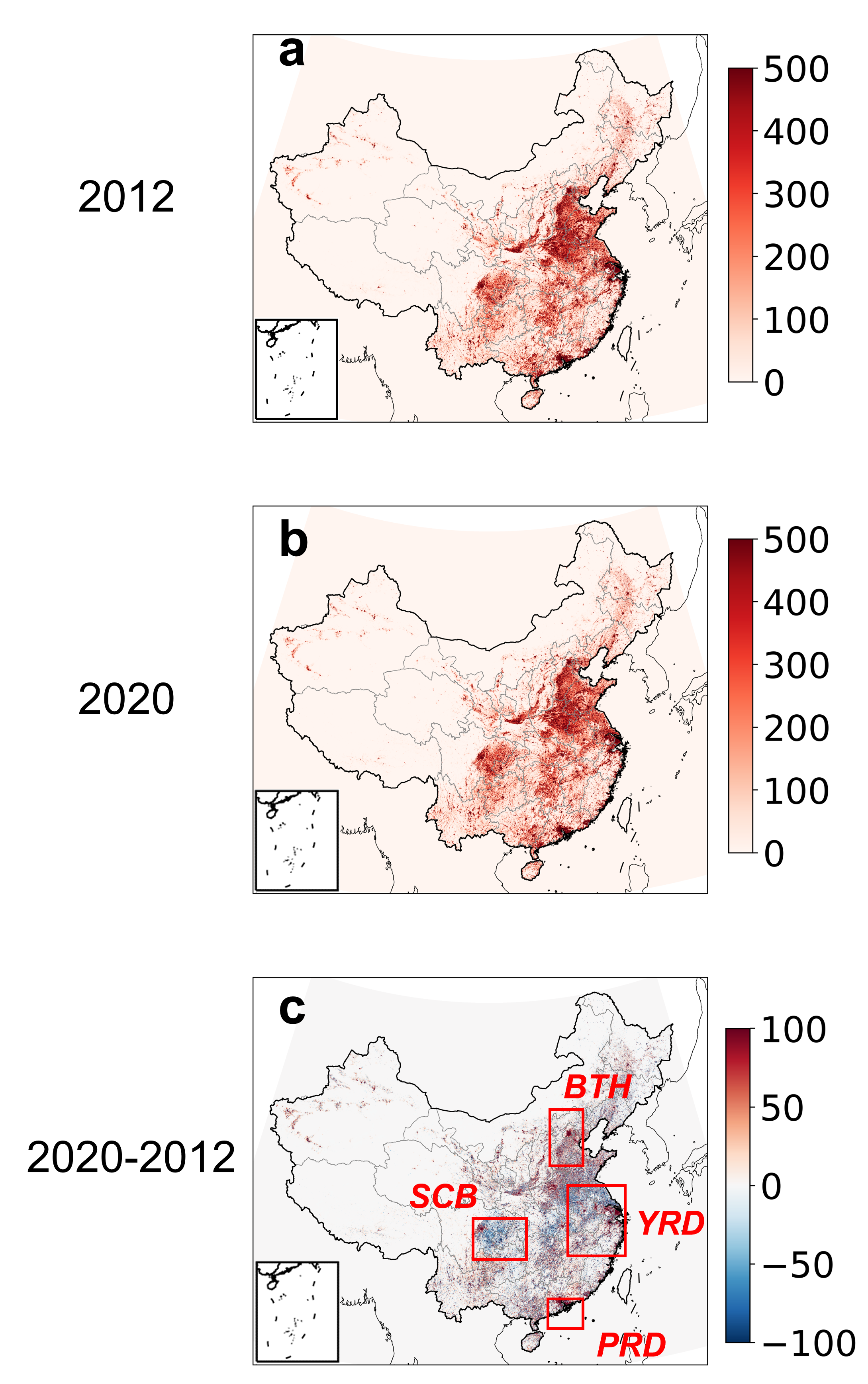
**

**Fig. S14.** Population Changes in China between 2012 and 2020, data were obtained from the WorldPop Hub Population Counts ([https://hub.worldpop.org/project/categories?id=3](https://hub.worldpop.org/project/categories?id=3), latest accessed on 09 December 2024). The authority number of the base map: GS (2022) 4314.

**
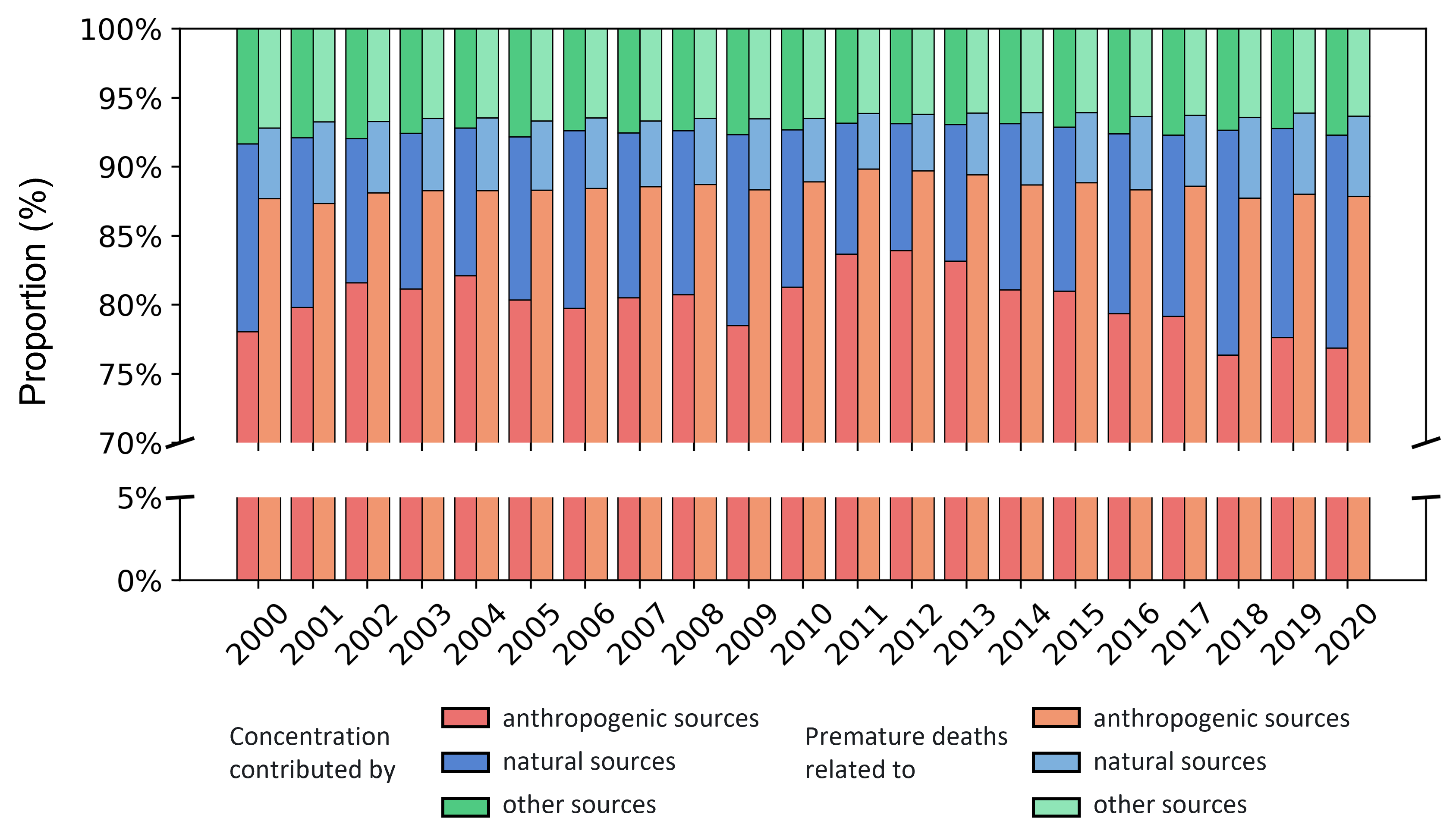
**

**Fig. S15.** Contribution ratios of anthropogenic sources, natural sources, and other sources to PM_2.5_ concentrations and related health risks in China from 2000 to 2020.

**References**

[1] A. B. Guenther, X. Jiang, C. L. Heald, T. Sakulyanontvittaya, T. a. Duhl, L. K. Emmons and X. Wang. The Model of Emissions of Gases and Aerosols from Nature version 2.1 (MEGAN2. 1): an extended and updated framework for modeling biogenic emissions. Geoscientific Model Development 5(6) (2012) 1471-1492.

[2] R. Burnett, H. Chen, M. Szyszkowicz, N. Fann, B. Hubbell, C. A. Pope, J. S. Apte, M. Brauer, A. Cohen, S. Weichenthal, J. Coggins, Q. Di, B. Brunekreef, J. Frostad, S. S. Lim, H. Kan, K. D. Walker, G. D. Thurston, R. B. Hayes, C. C. Lim, M. C. Turner, M. Jerrett, D. Krewski, S. M. Gapstur, W. R. Diver, B. Ostro, D. Goldberg, D. L. Crouse, R. V. Martin, P. Peters, L. Pinault, M. Tjepkema, A. van Donkelaar, P. J. Villeneuve, A. B. Miller, P. Yin, M. Zhou, L. Wang, N. A. H. Janssen, M. Marra, R. W. Atkinson, H. Tsang, T. Quoc Thach, J. B. Cannon, R. T. Allen, J. E. Hart, F. Laden, G. Cesaroni, F. Forastiere, G. Weinmayr, A. Jaensch, G. Nagel, H. Concin and J. V. Spadaro. Global estimates of mortality associated with long-term exposure to outdoor fine particulate matter. Proceedings of the National Academy of Sciences 115(38) (2018) 9592-9597.

[3] K. R. Daellenbach, G. Uzu, J. Jiang, L.-E. Cassagnes, Z. Leni, A. Vlachou, G. Stefenelli, F. Canonaco, S. Weber, A. Segers, J. J. P. Kuenen, M. Schaap, O. Favez, A. Albinet, S. Aksoyoglu, J. Dommen, U. Baltensperger, M. Geiser, I. El Haddad, J.-L. Jaffrezo and A. S. H. Prévôt. Sources of particulate-matter air pollution and its oxidative potential in Europe. Nature 587(7834) (2020) 414-419.

[4] C. Emery, E. Tai and G. Yarwood. Enhanced meteorological modeling and performance evaluation for two Texas ozone episodes. Prepared for the Texas natural resource conservation commission, by ENVIRON International Corporation (2001).

[5] U. Epa. Guidance on the use of models and other analyses for demonstrating attainment of air quality goals for ozone, PM2. 5, and regional haze. US Environmental Protection Agency, Office of Air Quality Planning and Standards (2007).

[6] X. Liu, X. Bai, H. Tian, K. Wang, S. Hua, H. Liu, S. Liu, B. Wu, Y. Wu, W. Liu, L. Luo, Y. Wang, J. Hao, S. Lin, S. Zhao and K. Zhang. Fine particulate matter pollution in North China: Seasonal-spatial variations, source apportionment, sector and regional transport contributions. Environmental Research 184 (2020) 109368.

[7] X. Li, Q. Zhang, Y. Zhang, B. Zheng, K. Wang, Y. Chen, T. J. Wallington, W. Han, W. Shen and X. Zhang. Source contributions of urban PM2. 5 in the Beijing–Tianjin–Hebei region: Changes between 2006 and 2013 and relative impacts of emissions and meteorology. Atmospheric Environment 123 (2015) 229-239.

[8] L. Shu, T. Wang, J. Liu, Z. Chen, H. Wu, Y. Qu, M. Li and M. Xie. Elucidating drivers of severe wintertime fine particulate matter pollution episodes in the Yangtze River Delta region of eastern China. Science of The Total Environment 912 (2024) 169546.

[9] Y. Xian, Y. Zhang, Z. Liu, H. Wang and T. Xiong. Characterization of winter PM2.5 source contributions and impacts of meteorological conditions and anthropogenic emission changes in the Sichuan Basin, 2002–2020. Science of The Total Environment 947 (2024) 174557.

[10] Z. Li, Y. Zhu, S. Wang, J. Xing, B. Zhao, S. Long, M. Li, W. Yang, R. Huang and Y. Chen. Source contribution analysis of PM2.5 using Response Surface Model and Particulate Source Apportionment Technology over the PRD region, China. Science of The Total Environment 818 (2022) 151757.

[11] X. Lu, Y. Chen, Y. Huang, C. Lin, Z. Li, J. C. H. Fung and A. K. H. Lau. Differences in concentration and source apportionment of PM2.5 between 2006 and 2015 over the PRD region in southern China. Science of The Total Environment 673 (2019) 708-718.

[12] L. Lv, P. Wei, J. Hu, Y. Chen and Y. Shi. Source apportionment and regional transport of PM2.5 during haze episodes in Beijing combined with multiple models. Atmospheric Research 266 (2022) 105957.

[13] X. Feng, Y. Feng, Y. Chen, J. Cai, Q. Li and J. Chen. Source apportionment of PM2.5 during haze episodes in Shanghai by the PMF model with PAHs. Journal of Cleaner Production 330 (2022) 129850.

[14] Y. Pan, Y. Zhu, J. Jang, S. Wang, J. Xing, P.-C. Chiang, X. Zhao, Z. You and Y. Yuan. Source and sectoral contribution analysis of PM2.5 based on efficient response surface modeling technique over Pearl River Delta Region of China. Science of The Total Environment 737 (2020) 139655.

[15] W. Zhang, B. Liu, Y. Zhang, Y. Li, X. Sun, Y. Gu, C. Dai, N. Li, C. Song, Q. Dai, Y. Han and Y. Feng. A refined source apportionment study of atmospheric PM2.5 during winter heating period in Shijiazhuang, China, using a receptor model coupled with a source-oriented model. Atmospheric Environment 222 (2020) 117157.

[16] M. Cheng, G. Tang, B. Lv, X. Li, X. Wu, Y. Wang and Y. Wang. Source apportionment of PM2.5 and visibility in Jinan, China. Journal of Environmental Sciences 102 (2021) 207-215.

[17] J. Mao, L. Yang, Z. Mo, Z. Jiang, P. Krishnan, S. Sarkar, Q. Zhang, W. Chen, B. Zhong and Y. Yang. Comparative study of chemical characterization and source apportionment of PM2. 5 in South China by filter-based and single particle analysis. Elem Sci Anth 9(1) (2021) 00046.

[18] J. Park, H. Kim, Y. Kim, J. Heo, S.-W. Kim, K. Jeon, S.-M. Yi and P. K. Hopke. Source apportionment of PM2. 5 in Seoul, South Korea and Beijing, China using dispersion normalized PMF. Science of The Total Environment 833 (2022) 155056.

[19] S. Kong, B. Han, Z. Bai, L. Chen, J. Shi and Z. Xu. Receptor modeling of PM2. 5, PM10 and TSP in different seasons and long-range transport analysis at a coastal site of Tianjin, China. Science of the Total Environment 408(20) (2010) 4681-4694.

[20] G. Shi, G. Chen, G. Liu, H. Wang, Y. Tian and Y. Feng. Source insights into the 11-h daytime and nighttime fine ambient particulate matter in China as well as the synthetic studies using the new Multilinear Engine 2-species ratios (ME2-SR) method. Journal of Environmental Management 181 (2016) 304-311.

[21] J. Gao, X. Peng, G. Chen, J. Xu, G.-L. Shi, Y.-C. Zhang and Y.-C. Feng. Insights into the chemical characterization and sources of PM2. 5 in Beijing at a 1-h time resolution. Science of the Total Environment 542 (2016) 162-171.

[22] C. Zhang, D. Jing, C. Wu, S. Li, N. Cheng, W. Li, G. Wang, B. Chen, Q. Wang and J. Hu. Integrating Chemical Mass Balance and the Community Multiscale Air Quality models for source identification and apportionment of PM2.5. Process Safety and Environmental Protection 149 (2021) 665-675.

[23] X. Qiao, Q. Ying, X. Li, H. Zhang, J. Hu, Y. Tang and X. Chen. Source apportionment of PM2.5 for 25 Chinese provincial capitals and municipalities using a source-oriented Community Multiscale Air Quality model. Science of The Total Environment 612 (2018) 462-471.

[24] X. Qiao, Y. Yuan, Y. Tang, Q. Ying, H. Guo, Y. Zhang and H. Zhang. Revealing the origin of fine particulate matter in the Sichuan Basin from a source-oriented modeling perspective. Atmospheric Environment 244 (2021) 117896.

[25] L. Li, J. Hu, J. Li, K. Gong, X. Wang, Q. Ying, M. Qin, H. Liao, S. Guo, M. Hu and Y. Zhang. Modelling air quality during the EXPLORE-YRD campaign – Part II. Regional source apportionment of ozone and PM2.5. Atmospheric Environment 247 (2021) 118063.

[26] J. Yang, S. Kang, Z. Ji, X. Chen, S. Yang, S.-Y. Lee, B. de Foy and D. Chen. A hybrid method for PM2.5 source apportionment through WRF-Chem simulations and an assessment of emission-reduction measures in western China. Atmospheric Research 236 (2020) 104787.

[27] Y. Li, L. Xue, Y. Tao, Y. Li, Y. Wu, Q. Liao, J. Wan and Y. Bai. Exploring the contributions of major emission sources to PM2.5 and attributable health burdens in China. Environmental Pollution 322 (2023) 121177.

[28] Y. Zhu, L. Huang, J. Li, Q. Ying, H. Zhang, X. Liu, H. Liao, N. Li, Z. Liu, Y. Mao, H. Fang and J. Hu. Sources of particulate matter in China: Insights from source apportionment studies published in 1987–2017. Environment International 115 (2018) 343-357.

[29] Q. Zhang, Y. Zheng, D. Tong, M. Shao, S. Wang, Y. Zhang, X. Xu, J. Wang, H. He and W. Liu. Drivers of improved PM2. 5 air quality in China from 2013 to 2017. Proceedings of the National Academy of Sciences 116(49) (2019) 24463-24469.

[30] G. Geng, Y. Liu, Y. Liu, S. Liu, J. Cheng, L. Yan, N. Wu, H. Hu, D. Tong, B. Zheng, Z. Yin, K. He and Q. Zhang. Efficacy of China’s clean air actions to tackle PM2.5 pollution between 2013 and 2020. Nature Geoscience (2024).
